# Impact of substance use disorders on critical care management and health outcomes in septic adolescents

**DOI:** 10.1186/s13613-025-01482-8

**Published:** 2025-05-10

**Authors:** Havell Markus, Gary D. Ceneviva, Neal J. Thomas, Conrad Krawiec

**Affiliations:** 1 Pennsylvania State University College of Medicine, 500 University Drive, P.O. Box 850, Hershey, PA 17033-0850 USA; 2 Program in Bioinformatics and Genomics, Pennsylvania State University, Huck Life Sciences Building, University Park, PA 16802 USA; 3 Medical Scientist Training Program, Pennsylvania State University College of Medicine, 500 University Drive, Hershey, PA 17033 USA; 4 Pediatric Critical Care Medicine, Department of Pediatrics, Penn State Hershey Children’s Hospital, 500 University Drive, P.O. Box 850, Hershey, PA 17033-0850 USA; 5 Department of Public Health Sciences, Pennsylvania State University College of Medicine, 500 University Drive, Hershey, PA 17033-0850 USA

**Keywords:** Substance use disorders, Adolescent, Young adult, Critical care, Electronic health records

## Abstract

**Background:**

Adult septic patients with substance abuse disorder (SUD) are at increased risk of poor outcomes, but the impact on adolescents is unknown. We aimed to determine if pre-existing SUD is associated with increased adverse outcomes and critical care resources in critically ill adolescents hospitalized with sepsis. We hypothesize that SUD is associated with increased risk of adverse outcomes and usage of critical care resources in this adolescent patient population.

**Methods:**

This was a retrospective cohort study utilizing the TriNetX© electronic health record (EHR) database, which consists of EHR from participating healthcare organizations predominantly in the United States. Critically ill adolescents with sepsis aged 12–21 years were divided into two groups (SUD history and no-SUD history). Data related to demographics, diagnostic, procedural, and medication codes were analyzed. The primary outcomes were organ dysfunction, critical care therapies, and all-cause 1-year mortality.

**Results:**

We included 5,436 critically ill adolescents with sepsis [730 (13.43%) SUD history and 4706 (86.57%) no-SUD history]. SUD history was associated with increased odds of organ dysfunction (adjusted odds ratio [aOR] 1.84; 95% confidence interval [CI] 1.56–2.16; *p* < 0.001), vasoactive/inotropic drug usage (aOR 1.29; 95% CI 1.10–1.52; *p* = 0.002), mechanical ventilation (aOR 2.19; 95% CI 1.85–2.59; *p* < 0.001), but not mortality (aOR 1.03; 95% CI 0.76–1.41; *p* = 0.83).

**Conclusions:**

Our retrospective analysis suggests history of SUD in critically ill septic adolescent patients is associated with increased utilization of critical care resources and organ dysfunction. Further study is needed to determine if substance abuse represents a potentially modifiable risk factor for critical illness in adolescent patients.

**Supplementary Information:**

The online version contains supplementary material available at 10.1186/s13613-025-01482-8.

## Key points

It is unknown whether substance abuse is a potentially modifiable risk factor for critical illness in adolescent patients. The findings from this retrospective study suggests history of substance use disorder in critically ill septic adolescent patients is associated with increased utilization of critical care resources and organ dysfunction. Thus, substance abuse may be a modifiable risk factor of adolescent health outcomes.

## Background

Despite being a leading cause of preventable morbidity and mortality, substance use disorder (SUD) among adolescents and young adults has increased at a rapid rate over the past 10 years [1–4]. SUD can begin as early as puberty and it is associated with poor quality of life and behavioral difficulties (e.g., hyperactivity/inattention, reduced prosocial behavior, conduct problems) [5]. Given the significant prevalence and impact of SUD in adolescents, it is considered a major societal concern.

SUD can have a significant direct effect on health. Hospitalized adult patients with SUD are more likely to be re-hospitalized within 30 days of discharge [6], to have worse proximal outcomes after discharge from treatment [7], and to die prematurely [8]. Specifically, patients with alcohol-use disorders undergoing surgeries experience higher rates of postoperative hemorrhage, cardiac complications, sepsis, and need for repeat surgeries [9–11]. In a recent report, patients with sepsis and opioid use disorder, compared with those without opioid use disorder, had lower mortality rates but had higher rates of infection, required more mechanical ventilation, experience more ICU admissions, spend more time in ICU, and spend more time in the hospital overall [12].

Although the burden of SUD on health conditions is widely recognized, most existing studies have solely focused on adult populations [6–11]. Additionally, while critically ill adult patients with SUD are impacted, it is unknown if the same effect is observed in adolescents. Various studies of critically ill adult patients with SUD suggest there is an urgent need to systematically and accurately identify patients with SUD, as overlooking potential existence of SUD in critically ill patients can lead to suboptimal care for both the underlying disease and substance use [9, 12, 13]. Such an investigation is vital to assess whether substance abuse represents a potentially modifiable risk factor for critical illness in adolescents.

The aim of our study is to determine whether history of SUD affects mortality, morbidity, and need for critical care resources in critically ill adolescent patients with sepsis through a retrospective analysis in a large nationwide electronic health record database. We hypothesize a pre-existing history of SUD is associated with increased adverse health outcomes and usage of critical care resources in critically ill adolescent patients hospitalized with sepsis.

## Methods

### Study design and data source

This is a retrospective observational cohort study examining the association of adverse health outcomes in patients with and without history of SUD in critically ill adolescents with sepsis. This study was conducted using TriNetX© electronic health record (EHR) data. TriNetX^®^ is a global federated health research network that provides researchers access to continuously updated data elements of every clinical encounter within the EHR from 173 participating healthcare organizations, predominantly in the United States and mostly consisting of large, academic medical centers, community hospitals, and outpatient clinics [14]. It encompasses of approximately 41.2 million pediatric patients aged ≤ 21 years old [15]. Thus, the data provided allows researchers to examine not only the time period of interest but also data before and after in a longitudinal fashion. TriNetX^®^ is certified to the ISO 27001:2013 standard and protects healthcare data by maintaining compliance with the Health Insurance Portability and Accountability Act (HIPPA) Security Rule. The EHR data elements are aggregated and de-identified, including demographic characteristics, diagnoses, procedures, medications, laboratory values, and genomics, all in compliance with the de-identification standard outlined in Section § 164.514(a) of the HIPPA privacy rule.

### Ethical considerations

Because no protected health information is received by the user, we were provided a waiver from the Penn State Health Institutional Review Board to perform this study (STUDY00020794).

### Data collection

On June 2nd, 2023, we analyzed the available EHR data of 5,436 subjects aged 12 to 21 defined by presence of both critical care billing codes and a sepsis associated diagnosis code [16]. We refer to our patient population as adolescents based on the refined adolescence definition (aged 10–24 years) by Sawyer et al. [17]. The timeframe of EHR data obtained was from 01/14/2008 to 05/24/2023. Index date was defined as the first occurrence of critical care billing codes occurring within seven days of a sepsis diagnostic code. The age of each subject was defined as the age at the index date. We excluded any subject that did not have an encounter any time before and up to 14 days before the index date, to ensure all subjects had at least one encounter before the index date to allow us to take advantage of the longitudinal features of this database.

After the initial query, the cohort was divided into two groups: subjects with and without history of SUD before the index date. Presence of International Classification of Diseases, 10th edition (ICD-10) diagnostic codes relating to SUD outlined by CMS Chronic Conditions Data Warehouse were used to define patients with history of SUD [18]. For each subject we obtained the following data: age, sex, race, regional location, ICD-10 diagnostic codes, medication codes, procedure codes, and deaths.

### Variables

We used ICD-10 codes prior to index date to determine if a subject had any social determinant of health (SDOH) if they had at least one occurrence of related ICD-10 codes. For pediatric complex chronic conditions (i.e. pediatric comorbidities), we utilized the R package pccc to identify these subjects [19]. We determined whether a subject had organ dysfunction based on the presence of ICD-10 codes within 7 days after the index date [20]. Previous studies show ICD-10 codes may be reliable proxies for assessing organ dysfunction severity, making them a useful alternative when other methodologies for assessing organ dysfunction, such as Sequential Organ Failure Assessment (SOFA) scores, are unavailable [21]. We further determined if a subject received any vasoactive treatments and mechanical ventilation within 7 days after the index date based on the presence of medications (as classified by the Anatomical Therapeutic Chemical Classification) and presence of procedures (as classified by the common procedural terminology (CPT), ICD-10-PCS, and healthcare common procedure coding system (HCPCS). We lastly defined whether a patient died within 1 year after the index date based on reported deaths in the database, which are sourced from in-patient death events and from mortality records from obituary databases and the Social Security Administration. Due to patient privacy reasons, the exact death dates were not provided by the TriNetX© database, only the month and year. To meet the definition of death within 1 year, we first added 365 days to the first recording of critical care services. If the month and year of the death date provided were within range of this calculation, then the patient met the definition of death within 365 days. For example, if the patient had received critical care services on January 1, 2020, the patient would not have been classified as a death within 365 days if the death date was reported as February 2023 but would have been if the death date was reported as December 2020. Because the data provided was deidentified, no date of birth was provided; therefore, ages are approximate. For example, a subject born in 2002 reported to have received critical care services on January 1, 2020 (i.e., day of catheter ablation), the subject was determined to be 18 years of age.

Please see Supplementary Table 1 for further details regarding diagnostic, procedure, and medication codes utilized in our outcome definitions.

### Statistical analysis

Given the history of SUD, we report the demographic characteristics and substance abuse summary for the included patients using summary statistics. For continuous characteristics (e.g., age) we report mean and standard deviation, and for categorical characteristics (e.g., presence of SDOH) we report total sample counts and proportions. We applied two sample t-test to compare continuous characteristics, and Chi-squared test and Fisher’s exact test to compare categorical characteristics between subjects with a SUD history and no SUD history.

We conducted univariate and multivariate logistic regression analysis to examine the association between various adverse health outcomes following the index date in critically ill adolescents with sepsis who have and do not have history of SUD. The primary outcomes analyzed were organ dysfunction within 7 days, usage of vasoactive/inotropic drugs within 7 days, administration of mechanical ventilation within 7 days, and patient death within 365 days. In the analysis, to improve model interpretability, we combined “Asian”, “American Indian or Alaska Native”, and “Native Hawaiian or Other Pacific Islander” in “race” to the “Other” category due to their relatively small proportions in the data. For each outcome, we first fit separate univariable logistic regression models for age, race, and presence of outcome, SDOH, and pediatric comorbidities to estimate unadjusted odds ratios (ORs) with 95% confidence intervals (CIs). We then fit a multivariate model that only included covariates that were significant in the univariate analysis (p-value < 0.05) to explore the independent effects after controlling for other factors. The OR for age represents the estimated change in the odds of the outcome variables per one-year increase in age alone (univariate) and age after controlling for other covariates (multivariate). We included age, race, SDOH, and pediatric comorbidities only as control variables to account for confounding effects and estimate a more accurate effect of different outcomes in critically ill septic adolescents with and without history of SUD. We did not anticipate any direct association between race and physiological response to sepsis.

Statistical software R v3.6.3 with packages readxl v1.3.1, dplyr v1.1.0, and tableone 0.13.2 were used to preprocess the data, conduct the statistical tests, and perform the logistic regression analyses. The default *glm()* function in R was used for the multivariate analysis, which employs iteratively reweighted least squares (IWLS) for finding the maximum likelihood estimates of the model parameters. P-values of less than or equal to 0.05 were regarded as statistically significant.

### Data availability

All data supporting the findings of this study are openly accessible in the published article and its supplementary information files. The raw EHR data can be obtained upon request. Data from the TriNetX© is available to licensed users.

## Results

### Demographics characteristics

A total of 5,436 critically ill subjects with sepsis [730 (13.43%) with SUD history and 4,706 (86.57%) with no-SUD history] were included in this study. Patients with a history of SUD were defined by the presence of ICD-10 codes for SUD prior to the index date (See Methods). Demographic characteristics and additional details are summarized in Table 1.

**Table 1:** Cohort demographic summary. The hypothesis test used for categorical variables is chi-square and for continuous variables is t-test. SUD = substance use disorder. SD = standard deviation. N = number of patients. % = percentage of patients

	No SUD	SUD	*p*-value
Total Subjects (n%)	4706 (86.57)	730 (13.43)	
Age (mean (SD))	17.07 (3.13)	19.01 (2.46)	< 0.001
Sex (n%)			0.002
Female	2508 (53.3)	344 (47.1)	
Male	2198 (46.7)	386 (52.9)	
Race (n%)			< 0.001
White	3242 (68.9)	576 (78.9)	
Black or African American	1268 (26.9)	132 (18.1)	
Asian	154 (3.3)	14 (1.9)	
American Indian or Alaska Native	30 (0.6)	5 (0.7)	
Native Hawaiian or Other Pacific Islander	12 (0.3)	3 (0.4)	
Social determinant of health (SDOH) diagnostic code presence (n%)	483 (10.3)	143 (19.6)	< 0.001
Pediatric Complex Clinical Conditions (n%)	2765 (58.8)	296 (40.5)	< 0.001
Congenital/Genetic Disorders (n%)	859 (18.3)	61 (8.4)	< 0.001
Cardiovascular (n%)	1065 (22.6)	156 (21.4)	0.477
Gastrointestinal (n%)	1036 (22.0)	90 (12.3)	< 0.001
Hematology/Immunology (n%)	717 (15.2)	74 (10.1)	< 0.001
Malignancy (n%)	558 (11.9)	58 (7.9)	0.002
Metabolic (n%)	1027 (21.8)	97 (13.3)	< 0.001
Neuromuscular (n%)	1094 (23.2)	88 (12.1)	< 0.001
Renal (n%)	640 (13.6)	59 (8.1)	< 0.001
Respiratory (n%)	404 (8.6)	35 (4.8)	0.001
Death within 1 year (n%)	341 (7.2)	54 (7.4)	0.944
Mechanical ventilation (n%)	1120 (23.8)	312 (42.7)	< 0.001
Organ dysfunction (n%)	2283 (48.5)	466 (63.8)	< 0.001
Respiratory failure (n%)	1819 (38.7)	392 (53.7)	< 0.001
Vasoactive (n%)	1404 (29.8)	262 (35.9)	0.001

### Patient substance abuse characteristics

Within the SUD group, cannabis related disorders were the most common, 51.5% of the subjects. The second most common category was the usage of other psychoactive substance and opioid related disorders, 33.8% and 30.0% of subjects respectively. Alcohol usage was present in 22.9% of subjects (Supplementary Table 2).

### Factors associated with adverse health outcomes in critically ill subjects with sepsis

History of SUD in critically ill subjects with sepsis was associated with a higher odd of organ dysfunction [OR: 1.84 (95% CI: 1.56–2.16), p-value < 0.001] (Table 2), usage of vasoactive/inotropic drugs [OR: 1.29 (95% CI: 1.10–1.52), p-value = 0.002] (Table 3), and mechanical ventilation [OR 2.19 (95% CI 1.85–2.59), p-value < 0.001] (Table 4) compared with patients without SUD history in multivariable analysis after controlling for sex, race, age, pediatric complex chronic conditions, and SDOH.

**Table 2:** Univariable and multivariable analysis for organ dysfunction in patients with and without history of SUD. SUD = substance use disorder. OR = Odds ratio. CI = confidence interval. SDOH = social determinants of health. Multivariable analysis includes covariates that are statistically significant in univariate analysis (p-value < 0.05).

	Univariable Analysis			Multivariable Analysis		
	**OR (95% CI)**	***P***-value	**OR (95% CI)**		***P***-value	
Sex (Ref: male)						
Female	0.64 (0.58–0.72)	***< 0.001***	0.65 (0.59–0.73)		***< 0.001***	
Race (Ref: White)						
Black or African American	1.07 (0.94–1.21)	0.30	-		-	
Other	0.85 (0.65–1.12)	0.25	-		-	
Age						
1-year increase	1.01 (0.99–1.03)	0.24	-		-	
Any pediatric complex chronic condition (Ref: Absence)						
Presence	0.91(0.82–1.01)	0.08	-		-	
SDOH (Ref: Absence)						
Presence	1.03 (0.87–1.22)	0.71	-		-	
SUD (Ref: Absence)						
Presence	1.87 (1.59–2.2)	***< 0.001***	1.84 (1.56–2.16)		***< 0.001***	

**Table 3:** Univariable and multivariable analysis for administration of vasoactive drugs in patients with and without history of SUD. SUD = substance use disorder. OR = Odds ratio. CI = confidence interval. SDOH = social determinants of health. Multivariable analysis includes covariates that are statistically significant in univariate analysis (p-value < 0.05)

	Univariable Analysis		Multivariable Analysis	
	**OR (95% CI)**	***P***-value	**OR (95% CI)**	***P***-value
Sex (Ref: male)				
Female	0.72 (0.64–0.81)	***< 0.001***	0.73 (0.65–0.82)	***< 0.001***
Race (Ref: White)				
Black or African American	1.03 (0.90–1.18)	0.64	-	-
Other	0.92 (0.68–1.25)	0.61	-	-
Age				
1-year increase	1.02 (1.00-1.04)	0.09	-	-
Any pediatric complex chronic condition (Ref: Absence)				
Presence	0.91 (0.81–1.02)	0.12	-	-
SDOH (Ref: Absence)				
Presence	1.10 (0.92–1.31)	0.30	-	-
SUD (Ref: Absence)				
Presence	1.32 (1.12–1.55)	***< 0.001***	1.29 (1.10–1.52)	***0.002***

**Table 4:** Univariable and multivariable analysis for mechanical ventilation in patients with and without history of SUD. SUD = substance use disorder. OR = Odds ratio. CI = confidence interval. SDOH = social determinants of health. Multivariable analysis includes covariates that are statistically significant in univariate analysis (p-value < 0.05)

	Univariable Analysis			Multivariable Analysis		
	**OR (95% CI)**	***P***-value	**OR (95% CI)**		***P***-value	
Sex (Ref: male)						
Female	0.57 (0.50–0.64)	***< 0.001***	0.57 (0.50–0.64)		***< 0.001***	
Race (Ref: White)						
Black or African American	0.95 (0.83–1.09)	0.49	*-*		-	
Other	0.91 (0.66–1.24)	0.54	*-*		-	
Age						
1-year increase	1.05 (1.03–1.07)	***< 0.001***	1.03 (1.01–1.05)		***0.006***	
Any pediatric complex chronic condition (Ref: Absence)						
Presence	0.88 (0.78–0.99)	***0.04***	0.94 (0.83–1.06)		0.29	
SDOH (Ref: Absence)						
Presence	1.08 (0.89–1.30)	0.44	*-*		-	
SUD (Ref: Absence)						
Presence	2.39 (2.03–2.81)	***< 0.001***	2.19 (1.85–2.59)		***< 0.001***	

Patients with SUD and no-SUD history had similar rates of death within 1 year after the index date, 7.2% and 7.4% respectively and mortality was not associated with SUD history in either univariable [OR: 1.02 (95% CI: 0.76–1.38), p-value = 0.88] or multivariable analysis [OR: 1.03 (95% CI: 0.76–1.41), p-value = 0.83] (Table 5).

**Table 5:** Univariable and multivariable analysis for death within 365 days in patients with and without history of SUD. SUD = substance use disorder. OR = Odds ratio. CI = confidence interval. SDOH = social determinants of health. Multivariable analysis includes covariates that are statistically significant in univariate analysis (p-value < 0.05)

	Univariable Analysis		Multivariable Analysis	
	**OR (95% CI)**	***P***-value	**OR (95% CI)**	***P***-value
Sex (Ref: male)				
Female	0.58 (0.47–0.72)	***< 0.001***	0.59 (0.48–0.73)	***< 0.001***
Race (Ref: White)				
Black or African American	0.88 (0.69–1.12)	0.31	-	-
Other	0.78 (0.44–1.38)	0.39	-	-
Age				
1-year increase	1.09 (1.05–1.13)	***< 0.001***	1.10 (1.06–1.14)	***< 0.001***
Any pediatric complex chronic condition (Ref: Absence)				
Presence	2.39 (1.90–3.02)	***< 0.001***	2.49 (1.96–3.15)	***< 0.001***
SDOH (Ref: Absence)				
Presence	0.59 (0.40–0.87)	***0.01***	0.58 (0.39–0.85)	***0.005***
SUD (Ref: Absence)				
Presence	1.02 (0.76–1.38)	0.88	1.03 (0.76–1.41)	0.83

Furthermore, combining “Asian,” “American Indian or Alaska Native,” and “Native Hawaiian or Other Pacific Islander” into an “Other” race category did not affect our results. As an example, sensitivity analysis, presented in Supplementary Table 3, for organ dysfunction confirmed this finding.

Lastly, in multivariate analysis, we observed female sex was associated with decreased odds of organ dysfunction [OR: 0.65 (95% CI: 0.59–0.73, *p* < 0.001] (Table 2), vasoactive/inotropic drug usage [OR: 0.73 (95% CI: 0.65–0.82, *p* < 0.001] (Table 3), mechanical ventilation [OR: 0.57 (95% CI: 0.50–0.64), *p* < 0.001], and mortality [OR: 0.59 (95% CI: 0.48–0.73), *p* < 0.001].

## Discussions

In our present study, we aimed to determine the effect of a pre-existing SUD in critically ill adolescent patients with sepsis by measuring various health outcomes related to morbidity and mortality. Consistent with our hypothesis, we observed that critically ill adolescents with sepsis who have a SUD history had a higher odds of organ dysfunction, usage of vasoactive/inotropic drugs, and mechanical ventilation when compared to adolescents without SUD history. Despite the observed associations with increased morbidity and requirement for critical care therapies, patients with and without SUD history had similar all-cause mortality within 365 days after the index date. Our findings identify a history of SUD as a novel risk factor for increased morbidity and usage of critical care resources in critically ill adolescent patients with sepsis. To our knowledge, this is the first and largest population study of critically ill septic adolescents on this topic, advancing our understanding of impact of SUD in this population.

Although not a primary aim of our study, we observed female sex was associated with decreased utilization of critical care resources, organ dysfunction, and mortality rates in critically ill adolescents with sepsis. This sex difference in sepsis outcomes has been extensively reported and is attributed to a combination of biological and social factors, such as sex hormone variations, gene expression from the X chromosome, and sociocultural influences [22–26].

SUD among adults and children is on the rise and further increased due to the COVID-19 pandemic [27–29]. According to the 2021 Monitoring the Future (MTF) nationwide surveys, 10.2%, 18.7%, and 32% of 8th, 10th, and 12th graders reported using at least one illicit drug within the past year [1, 30]. SUD can have a long-lasting impact on the life of children and adolescents, as it is associated with higher underachievement in school, delinquency, teenage pregnancy, and depression [3, 31, 32]. Thus, identifying at-risk individuals through proper screening and making efforts to prevent or reduce SUD in this population should be prioritized during all clinical encounters. However, despite existence of various evidence-based tools or strategies for screening and providing interventions or treatments [33, 34], many adolescent patients with SUD do not receive this crucial intervention due to two reasons. First, various studies suggest that physicians fail to screen for SUD in adolescents due to lack of time, lack of training, or discomfort, which impedes delivery of preventative care [35, 36]. This also suggests SUD in adolescent patient population is under documented in health records. Second, many adolescents at risk for substance use or have a history SUD may not attend routine primary care visits and rely on hospital-based setting to receive health care [37–39]. Pediatric critical care clinicians may see more adolescent patients with SUD and are presented with a unique opportunity to systematically screening for SUD and offer proper intervention and treatment.

Understanding how SUD can impact health outcomes in critically ill adolescent patients could be essential for appropriately planning management strategies and minimizing the overall cost of expensive and complex multiorgan care. SUD has been reported to have direct negative health outcomes in adults. For example, opioid usage or overdose could lead to respiratory suppression/failure [40, 41], immunosuppression, impairment of gut barrier integrity, and modulation of gut microbiota [42, 43]. Chronic usage of tobacco, alcohol, and other drugs are associated with cardiovascular (myocardial infraction, arrythmias, cardiac insufficiency), pulmonary (COPD, pulmonary hypertension), and metabolic (diabetes, hypertension) diseases [44–47]. Smoking and SUD are among the conditions associated with high risk of severe COVID-19 illness by Centers for Disease Control and Prevention [47]. Furthermore, the health effects of SUD are enhanced in critically ill adult patients, as history of SUD could have significant influence on morbidity, mortality, and usage of critical care resources [5, 6, 8–12]. However, despite the increasing prevalence of SUD among both adults and adolescents, little is known about the impact of SUD in critically ill adolescent patients. Thus, through improved systematic screening for SUD and knowing whether SUD impacts adolescents similarly to adults could inform unique strategies to (1) clinically manage these high-risk patients and (2) address overall SUD in adolescents through implementation of interventions for prevention and cessation of substance use.

In our analysis, we observed that pre-existing SUD have largely similar effects in adolescents as reported in adults. We first observed critically ill septic adolescents with SUD history were associated with a higher odds of organ dysfunction, usage of vasoactive/inotropic drugs, and mechanical ventilation when compared to patients without SUD history. This suggests the severity of sepsis could be higher in patients with SUD history and requires more aggressive care for management, incurring a higher cost of care. In line with our results, a previous study of pediatric opioid-related hospitalization observed 42.9% required PICU care and among the PICU admissions 37% required mechanical ventilation support and 20.3% required vasopressors [48]. As mentioned, previous studies have reported chronic usage of various drugs could directly lead to respiratory suppression and effect multiple organ systems, which could explain higher rates of organ dysfunction and usage of vasoactive/inotropic drugs and mechanical ventilation.

Despite higher rates of organ dysfunction, usage of vasoactive/inotropic drugs, and mechanical ventilation, we did not observe differences in death within 365 days after the index date between groups. This finding has also been observed in adult patients. Adult patients with sepsis and opioid use disorder, compared with those without opioid use disorder, had lower mortality rates but had higher rates of infection and required more mechanical ventilation, experience more ICU admissions, spend more time in ICU, and spend more time in the hospital overall [12]. The authors of that study postulated they did not observe a difference in mortality potentially due to some degree of residual confounding that might be mediated by difference in age and presence of chronic conditions. Such reasoning could also be applied to our observations. Another potential reason could be that adolescent health care system has improved over the years with overall reduced mortality over time. A previous study of pediatric opioid-related hospitalization observed the annual deaths significantly decreased from 2.8 to 1.3% between 2004 and 2007 and 2012–2015 periods [48]. Another potential reason for these findings could be due to a lack of power, which would require a larger cohort to capture differences.

### Strengths and limitations

Strengths of this study include (1) the large sample size (*n* = 5,436), (2) retrospective observational design was efficient and cost effective, and (3) TriNetX^®^ EHR pulled data from multiple institutions throughout United States allowing for good external validity.

There are several limitations to this study. While our analysis focused on general SUD, we did not differentiate the physiological effects of different substances (e.g., alcohol, nicotine, cannabis, opioids, etc.). Future studies with larger sample sizes and more detailed substance use assessments may elucidate how specific substances might uniquely influence sepsis outcomes. This was a retrospective study, thus the associations we found are not causation. Due to the limitations of the database, clinical notes from the providers were not available for review. Therefore, we do not know how each patient presented in clinic. Our data was also restricted to institutions that participate in this database retrieval system; thus, we are limited in the patient data available. We depended on clinicians to accurately enter diagnostic, procedure, or medication codes to infer whether a subject had a given clinical feature. It is possible that not all EHR data were reported for a subject or not all subjects who had sepsis, SUD, or other clinical features were coded accurately in the EHR database. As such, given that the TriNetX^®^ is a generalist database, it may not provide the level of granularity or control offered by specialized data sources. Although, studies from such generalist databases are still valuable for identifying patterns and generating clinically relevant hypothesis, conclusions drawn from our study should be interpreted with caution and require further studies using targeted datasets or methodologies to validate our findings. We identified pediatric sepsis and organ dysfunction cases using diagnostic codes, but several factors may limit the accuracy and generalizability of our findings. First, changes in the definition of pediatric sepsis and EHR coding practices over the past decade could affect results. Second, while ICD-10 codes have high specificity (~ 98%) for sepsis, they have low sensitivity (~ 35%) [49]. Finally, some studies suggest that ICD codes may capture more severely ill septic patients [50]. Thus, our cohort likely represents a subset of severe sepsis cases, potentially introducing ascertainment bias and limiting the generalizability of our findings. Future studies using validated methods to capture pediatric sepsis are needed to better assess this bias.

## Conclusion

SUD is a major health and societal concern in adolescents. Our results suggest that although history of SUD does not impact mortality, it could increase severity of illness in critically ill adolescents with sepsis, requiring increased usage of aggressive treatments and utilization of critical care resources for management. In line with our results, we believe improved and increased screening for history of substance use during hospitalization of critically ill adolescents who are at risk of sepsis may enable early identification of patients with potentially worse clinical course and referral of patients who can benefit from interventions for prevention and cessation of substance use. In addition, we believe such changes would potentially decrease the overall financial burden on patients or their families and may recuperate the overall hospital-associated costs related to care of severely ill patients.

## Electronic supplementary material

Below is the link to the electronic supplementary material.

Supplementary Material 1

## Abbreviations

SUD: substance abuse disorder
EHR: electronic health record
CI: confidence interval
OR: odds ratio
aOR: adjusted odds ratio
SD: standard deviation
HIPPA: Health Insurance Portability and Accountability Act
ICD: International Classification of Diseases
CPT: common procedural terminology
HCPCS: healthcare common procedure coding system
SDOH: social determinant of health
