# Supplementary material for: Impact of substance use disorders on critical care management and health outcomes in septic adolescents
Source: Ann Intensive Care. 2025 May 10;15:63. doi: 10.1186/s13613-025-01482-8 (PMC12065691; doi:10.1186/s13613-025-01482-8)
Supplement: Supplementary file 1 — Supplementary Material 1 [file 13613_2025_1482_MOESM1_ESM.docx]

**Impact of substance use disorders on critical care management and health outcomes in septic adolescents and young adults**

**Supplementary Tables**

**Table of content:**

| Supplementary Table 1: Diagnostic, procedural, and medication codes utilized to define various clinical features in this study. | Page 3-20 |
| --- | --- |
| Supplementary Table 2: Patient substance abuse summary. | Page 21 |
| Supplementary Table 3: Univariable and multivariable analysis for organ dysfunction in patients with and without history of SUD. | Page 22 |

**Supplementary Table 1: Diagnostic, procedural, and medication codes utilized to define various clinical features in this study.** Diagnostic [International Classification of Diseases, 9^th^ (ICD-9-CM) and 10^th^ edition (ICD-10-CM)]; Clinical Modification and Procedural [International Classification of Diseases Procedural Coding System, 10^th^ edition (ICD-10-PCS); Common Procedural Terminology (CPT); and Healthcare Common Procedure Coding System (HCPCS)]; Medication Code [RxNorm]; National Drug Code [NDC]

| **Category** | **Description** |
| --- | --- |
| **Alcohol Abuse** | "291.0" (ICD-9-CM: "Alcohol withdrawal delirium"); "305.00" (ICD-9-CM: "Alcohol abuse, unspecified"); "V65.42" (ICD-9-CM: "Counseling on substance use and abuse"); "291.89" (ICD-9-CM: "Other alcohol-induced mental disorders"); "305.01" (ICD-9-CM: "Alcohol abuse, continuous"); "291.81" (ICD-9-CM: "Alcohol withdrawal"); "303.90" (ICD-9-CM: "Other and unspecified alcohol dependence, unspecified"); "571.2" (ICD-9-CM: "Alcoholic cirrhosis of liver"); "303.01" (ICD-9-CM: "Acute alcoholic intoxication in alcoholism, continuous"); "291.9" (ICD-9-CM: "Unspecified alcohol-induced mental disorders"); "303.00" (ICD-9-CM: "Acute alcoholic intoxication in alcoholism, unspecified"); "980.0" (ICD-9-CM: "Toxic effect of ethyl alcohol"); "305.02" (ICD-9-CM: "Alcohol abuse, episodic"); "303.92" (ICD-9-CM: "Other and unspecified alcohol dependence, episodic"); "760.71" (ICD-9-CM: "Alcohol affecting fetus or newborn via placenta or breast milk"); "571.1" (ICD-9-CM: "Acute alcoholic hepatitis"); "V79.1" (ICD-9-CM: "Screening for alcoholism"); "571.3" (ICD-9-CM: "Alcoholic liver damage, unspecified"); "E860.0" (ICD-9-CM: "Accidental poisoning by alcoholic beverages"); "535.30" (ICD-9-CM: "Alcoholic gastritis, without mention of hemorrhage"); "535.31" (ICD-9-CM: "Alcoholic gastritis, with hemorrhage"); "303.91" (ICD-9-CM: "Other and unspecified alcohol dependence, continuous"); "303.02" (ICD-9-CM: "Acute alcoholic intoxication in alcoholism, episodic"); "291.4" (ICD-9-CM: "Idiosyncratic alcohol intoxication"); "291.2" (ICD-9-CM: "Alcohol-induced persisting dementia"); "425.5" (ICD-9-CM: "Alcoholic cardiomyopathy"); "291.3" (ICD-9-CM: "Alcohol-induced psychotic disorder with hallucinations"); "291.8" (ICD-9-CM: "Other specified alcohol-induced mental disorders"); "571.0" (ICD-9-CM: "Alcoholic fatty liver"); "291.5" (ICD-9-CM: "Alcohol-induced psychotic disorder with delusions"); "291.82" (ICD-9-CM: "Alcohol induced sleep disorders"); "F10.129" (ICD-10-CM: "Alcohol abuse with intoxication, unspecified"); "F10.10" (ICD-10-CM: "Alcohol abuse, uncomplicated"); "T51.0X1A" (ICD-10-CM: "Toxic effect of ethanol, accidental (unintentional), initial encounter"); "F10.920" (ICD-10-CM: "Alcohol use, unspecified with intoxication, uncomplicated"); "F10.19" (ICD-10-CM: "Alcohol abuse with unspecified alcohol-induced disorder"); "F10.231" (ICD-10-CM: "Alcohol dependence with withdrawal delirium"); "F10.929" (ICD-10-CM: "Alcohol use, unspecified with intoxication, unspecified"); "F10.930" (ICD-10-CM: "Alcohol use, unspecified with withdrawal, uncomplicated"); "F10.932" (ICD-10-CM: "Alcohol use, unspecified with withdrawal with perceptual disturbance"); "G62.1" (ICD-10-CM: "Alcoholic polyneuropathy"); "K70.10" (ICD-10-CM: "Alcoholic hepatitis without ascites"); "Q86.0" (ICD-10-CM: "Fetal alcohol syndrome (dysmorphic)"); "F10.959" (ICD-10-CM: "Alcohol use, unspecified with alcohol-induced psychotic disorder, unspecified"); "T51.0X2A" (ICD-10-CM: "Toxic effect of ethanol, intentional self-harm, initial encounter"); "F10.120" (ICD-10-CM: "Alcohol abuse with intoxication, uncomplicated"); "F10.20" (ICD-10-CM: "Alcohol dependence, uncomplicated"); "F10.94" (ICD-10-CM: "Alcohol use, unspecified with alcohol-induced mood disorder"); "F10.229" (ICD-10-CM: "Alcohol dependence with intoxication, unspecified"); "F10.99" (ICD-10-CM: "Alcohol use, unspecified with unspecified alcohol-induced disorder"); "Z71.41" (ICD-10-CM: "Alcohol abuse counseling and surveillance of alcoholic"); "F10.921" (ICD-10-CM: "Alcohol use, unspecified with intoxication delirium"); "F10.239" (ICD-10-CM: "Alcohol dependence with withdrawal, unspecified"); "K29.20" (ICD-10-CM: "Alcoholic gastritis without bleeding"); "F10.220" (ICD-10-CM: "Alcohol dependence with intoxication, uncomplicated"); "F10.121" (ICD-10-CM: "Alcohol abuse with intoxication delirium"); "F10.230" (ICD-10-CM: "Alcohol dependence with withdrawal, uncomplicated"); "K70.31" (ICD-10-CM: "Alcoholic cirrhosis of liver with ascites"); "F10.139" (ICD-10-CM: "Alcohol abuse with withdrawal, unspecified"); "F10.14" (ICD-10-CM: "Alcohol abuse with alcohol-induced mood disorder"); "T51.0X4A" (ICD-10-CM: "Toxic effect of ethanol, undetermined, initial encounter"); "F10.24" (ICD-10-CM: "Alcohol dependence with alcohol-induced mood disorder"); "F10.951" (ICD-10-CM: "Alcohol use, unspecified with alcohol-induced psychotic disorder with hallucinations"); "F10.130" (ICD-10-CM: "Alcohol abuse with withdrawal, uncomplicated"); "F10.931" (ICD-10-CM: "Alcohol use, unspecified with withdrawal delirium"); "F10.939" (ICD-10-CM: "Alcohol use, unspecified with withdrawal, unspecified"); "K70.30" (ICD-10-CM: "Alcoholic cirrhosis of liver without ascites"); "F10.221" (ICD-10-CM: "Alcohol dependence with intoxication delirium"); "F10.29" (ICD-10-CM: "Alcohol dependence with unspecified alcohol-induced disorder"); "K70.40" (ICD-10-CM: "Alcoholic hepatic failure without coma"); "F10.280" (ICD-10-CM: "Alcohol dependence with alcohol-induced anxiety disorder"); "F10.988" (ICD-10-CM: "Alcohol use, unspecified with other alcohol-induced disorder"); "I42.6" (ICD-10-CM: "Alcoholic cardiomyopathy"); "K29.21" (ICD-10-CM: "Alcoholic gastritis with bleeding"); "K70.11" (ICD-10-CM: "Alcoholic hepatitis with ascites"); "F10.188" (ICD-10-CM: "Alcohol abuse with other alcohol-induced disorder"); "K70.9" (ICD-10-CM: "Alcoholic liver disease, unspecified"); "F10.980" (ICD-10-CM: "Alcohol use, unspecified with alcohol-induced anxiety disorder"); "F10.151" (ICD-10-CM: "Alcohol abuse with alcohol-induced psychotic disorder with hallucinations"); "F10.132" (ICD-10-CM: "Alcohol abuse with withdrawal with perceptual disturbance"); "F10.982" (ICD-10-CM: "Alcohol use, unspecified with alcohol-induced sleep disorder"); "F10.232" (ICD-10-CM: "Alcohol dependence with withdrawal with perceptual disturbance"); "K70.41" (ICD-10-CM: "Alcoholic hepatic failure with coma"); "F10.288" (ICD-10-CM: "Alcohol dependence with other alcohol-induced disorder"); "F10.259" (ICD-10-CM: "Alcohol dependence with alcohol-induced psychotic disorder, unspecified"); "K70.0" (ICD-10-CM: "Alcoholic fatty liver"); "F10.159" (ICD-10-CM: "Alcohol abuse with alcohol-induced psychotic disorder, unspecified"); "F10.180" (ICD-10-CM: "Alcohol abuse with alcohol-induced anxiety disorder"); "F10.96" (ICD-10-CM: "Alcohol use, unspecified with alcohol-induced persisting amnestic disorder"); "T51.0X3A" (ICD-10-CM: "Toxic effect of ethanol, assault, initial encounter"); "F10.251" (ICD-10-CM: "Alcohol dependence with alcohol-induced psychotic disorder with hallucinations"); "F10.950" (ICD-10-CM: "Alcohol use, unspecified with alcohol-induced psychotic disorder with delusions"); |
| **Cardiovascular Medications** | "3992" (RxNorm: "epinephrine"); "8163" (RxNorm: "phenylephrine"); "7512" (RxNorm: "norepinephrine"); "3628" (RxNorm: "dopamine"); "3616" (RxNorm: "dobutamine"); "11149" (RxNorm: "vasopressin (USP)"); "1010751" (RxNorm: "epinephrine"); "1660014" (RxNorm: "epinephrine"); "1234579" (RxNorm: "phenylephrine"); "310132" (RxNorm: "epinephrine"); "1870207" (RxNorm: "epinephrine"); "309986" (RxNorm: "dobutamine"); "1010759" (RxNorm: "epinephrine"); "242969" (RxNorm: "norepinephrine"); "1012381" (RxNorm: "epinephrine"); "1666372" (RxNorm: "phenylephrine"); "1870230" (RxNorm: "epinephrine"); "727373" (RxNorm: "epinephrine"); "1012384" (RxNorm: "epinephrine"); "1672919" (RxNorm: "epinephrine"); "313578" (RxNorm: "vasopressin (USP)"); "209217" (RxNorm: "norepinephrine"); "2103182" (RxNorm: "vasopressin (USP)"); "1010745" (RxNorm: "epinephrine"); "1298068" (RxNorm: "phenylephrine"); "1298360" (RxNorm: "phenylephrine"); "1232651" (RxNorm: "phenylephrine"); "1867596" (RxNorm: "epinephrine"); "2475340" (RxNorm: "norepinephrine"); "309987" (RxNorm: "dobutamine"); "1293648" (RxNorm: "epinephrine"); "2619579" (RxNorm: "norepinephrine"); "1010688" (RxNorm: "epinephrine"); "2475337" (RxNorm: "norepinephrine"); "409175250" (NDC: "epinephrine"); "42023015901" (NDC: "epinephrine"); "1660016" (RxNorm: "epinephrine"); "641614225" (NDC: "phenylephrine"); "409120901" (NDC: "epinephrine"); "1010755" (RxNorm: "epinephrine"); "727347" (RxNorm: "epinephrine"); "1867940" (RxNorm: "epinephrine"); "1010763" (RxNorm: "epinephrine"); "1867993" (RxNorm: "epinephrine"); "1010692" (RxNorm: "epinephrine"); "1305269" (RxNorm: "epinephrine"); "76329331601" (NDC: "epinephrine"); "409724125" (NDC: "epinephrine"); "409174610" (NDC: "epinephrine"); "1867612" (RxNorm: "epinephrine"); "49502050002" (NDC: "epinephrine"); "76329906100" (NDC: "epinephrine"); "1012413" (RxNorm: "epinephrine"); "2533437" (RxNorm: "phenylephrine"); "2103184" (RxNorm: "vasopressin (USP)"); "1012461" (RxNorm: "epinephrine"); "1867594" (RxNorm: "epinephrine"); "1012377" (RxNorm: "epinephrine"); "1867998" (RxNorm: "epinephrine"); "1490057" (RxNorm: "epinephrine"); "1743877" (RxNorm: "dopamine"); "1672917" (RxNorm: "epinephrine"); "1870205" (RxNorm: "epinephrine"); "338100902" (NDC: "dopamine"); "1292887" (RxNorm: "dopamine"); "76014000410" (NDC: "phenylephrine"); "310116" (RxNorm: "epinephrine"); "1593738" (RxNorm: "vasopressin (USP)"); "313967" (RxNorm: "epinephrine"); "409781022" (NDC: "dopamine"); "42023016425" (NDC: "vasopressin (USP)"); "63323048227" (NDC: "epinephrine"); "1113705" (RxNorm: "phenylephrine"); "409724110" (NDC: "epinephrine"); "42023015925" (NDC: "epinephrine"); "727386" (RxNorm: "epinephrine"); "1867938" (RxNorm: "epinephrine"); "1867943" (RxNorm: "epinephrine"); "1743879" (RxNorm: "dopamine"); "63323030201" (NDC: "vasopressin (USP)"); "1743938" (RxNorm: "dopamine"); "63323046237" (NDC: "epinephrine"); "63323048917" (NDC: "epinephrine"); "54288010310" (NDC: "epinephrine"); "63323048927" (NDC: "epinephrine"); "409317802" (NDC: "epinephrine"); "409904217" (NDC: "epinephrine"); "409317801" (NDC: "epinephrine"); "409337504" (NDC: "norepinephrine"); "36000016210" (NDC: "norepinephrine"); "1232653" (RxNorm: "phenylephrine"); "1867614" (RxNorm: "epinephrine"); "1867618" (RxNorm: "epinephrine"); "1867620" (RxNorm: "epinephrine"); "1010749" (RxNorm: "epinephrine"); "76014000425" (NDC: "phenylephrine"); "409724101" (NDC: "epinephrine"); "409317701" (NDC: "epinephrine"); "409144304" (NDC: "norepinephrine"); "409910420" (NDC: "dopamine"); "517102025" (NDC: "vasopressin (USP)"); "409904201" (NDC: "epinephrine"); "1427404" (RxNorm: "phenylephrine"); "63323048731" (NDC: "epinephrine"); "63323046157" (NDC: "epinephrine"); "1743953" (RxNorm: "dopamine"); "1812168" (RxNorm: "dobutamine"); "1234569" (RxNorm: "phenylephrine"); "1867992" (RxNorm: "epinephrine"); "309985" (RxNorm: "dobutamine"); "1114874" (RxNorm: "dopamine"); "2591431" (RxNorm: "vasopressin (USP)"); "63323046837" (NDC: "epinephrine"); "703115303" (NDC: "norepinephrine"); "409724161" (NDC: "epinephrine"); "409492134" (NDC: "epinephrine"); "76329906000" (NDC: "epinephrine"); "1234584" (RxNorm: "phenylephrine"); "409904202" (NDC: "epinephrine"); "891438" (RxNorm: "epinephrine"); "827706" (RxNorm: "phenylephrine"); "1234563" (RxNorm: "phenylephrine"); "1234578" (RxNorm: "phenylephrine"); "727316" (RxNorm: "epinephrine"); "409318201" (NDC: "epinephrine"); "1369842" (RxNorm: "phenylephrine"); "409337525" (NDC: "norepinephrine"); "409904301" (NDC: "epinephrine"); "63323048737" (NDC: "epinephrine"); "641614201" (NDC: "phenylephrine"); "1087047" (RxNorm: "phenylephrine"); "1595029" (RxNorm: "epinephrine"); "1234585" (RxNorm: "phenylephrine"); "2591428" (RxNorm: "vasopressin (USP)"); "409234733" (NDC: "dobutamine"); "409174929" (NDC: "epinephrine"); "409317818" (NDC: "epinephrine"); "409904211" (NDC: "epinephrine"); "409317803" (NDC: "epinephrine"); "1011809" (RxNorm: "epinephrine"); "1299141" (RxNorm: "phenylephrine"); "67457085204" (NDC: "norepinephrine"); "409780922" (NDC: "dopamine"); "1300092" (RxNorm: "phenylephrine"); "1542385" (RxNorm: "phenylephrine"); "1666374" (RxNorm: "phenylephrine"); "50991074001" (NDC: "phenylephrine"); "2172190" (RxNorm: "phenylephrine"); "1867996" (RxNorm: "epinephrine"); "1867997" (RxNorm: "epinephrine"); "1867999" (RxNorm: "epinephrine"); "1234581" (RxNorm: "phenylephrine"); "1234576" (RxNorm: "phenylephrine"); "1234586" (RxNorm: "phenylephrine"); "409493301" (NDC: "epinephrine"); "42023016899" (NDC: "epinephrine"); "42023021901" (NDC: "vasopressin (USP)"); "2399909" (RxNorm: "vasopressin (USP)"); "2399907" (RxNorm: "vasopressin (USP)"); "63323046357" (NDC: "epinephrine"); "1012417" (RxNorm: "epinephrine"); "42023016801" (NDC: "epinephrine"); "63323046817" (NDC: "epinephrine"); "63323048217" (NDC: "epinephrine"); "409904517" (NDC: "epinephrine"); "143931801" (NDC: "norepinephrine"); "1743941" (RxNorm: "dopamine"); "42023019001" (NDC: "vasopressin (USP)"); "63323048257" (NDC: "epinephrine"); "49502010202" (NDC: "epinephrine"); "338107702" (NDC: "dobutamine"); "781922870" (NDC: "phenylephrine"); "70121157601" (NDC: "norepinephrine"); "409781011" (NDC: "dopamine"); "409904601" (NDC: "epinephrine"); "1867944" (RxNorm: "epinephrine"); "63323048921" (NDC: "epinephrine"); "50991078260" (NDC: "phenylephrine"); "2262290" (RxNorm: "phenylephrine"); "1049182" (RxNorm: "phenylephrine"); "2262018" (RxNorm: "phenylephrine"); "2533434" (RxNorm: "phenylephrine"); "42023016410" (NDC: "vasopressin (USP)"); "63323075101" (NDC: "phenylephrine"); "338100702" (NDC: "dopamine"); "781375595" (NDC: "norepinephrine"); "1657026" (RxNorm: "epinephrine"); "1670365" (RxNorm: "epinephrine"); "338100703" (NDC: "dopamine"); "1868028" (RxNorm: "epinephrine"); "1868029" (RxNorm: "epinephrine"); "42023016401" (NDC: "vasopressin (USP)"); "1356789" (RxNorm: "phenylephrine"); "1251022" (RxNorm: "phenylephrine"); "1299137" (RxNorm: "phenylephrine"); "42702010215" (NDC: "phenylephrine"); "54288010301" (NDC: "epinephrine"); "63323048157" (NDC: "epinephrine"); "409174630" (NDC: "epinephrine"); "409318202" (NDC: "epinephrine"); "409904502" (NDC: "epinephrine"); "63323048792" (NDC: "epinephrine"); "76014000433" (NDC: "phenylephrine"); "1549386" (RxNorm: "phenylephrine"); "238996" (RxNorm: "phenylephrine"); "409174910" (NDC: "epinephrine"); "409234632" (NDC: "dobutamine"); "42023023710" (NDC: "vasopressin (USP)"); "63323075105" (NDC: "phenylephrine"); "115169449" (NDC: "epinephrine"); "1372312" (RxNorm: "phenylephrine"); "1011820" (RxNorm: "epinephrine"); "63323046301" (NDC: "epinephrine"); "573287110" (NDC: "phenylephrine"); "63323048205" (NDC: "epinephrine"); "409318301" (NDC: "epinephrine"); "536138912" (NDC: "phenylephrine"); "312398" (RxNorm: "phenylephrine"); "31382089805" (NDC: "epinephrine"); "67208601" (NDC: "phenylephrine"); "363064810" (NDC: "phenylephrine"); "536118612" (NDC: "phenylephrine"); "1438497" (RxNorm: "phenylephrine"); "338107502" (NDC: "dobutamine"); "338011220" (NDC: "norepinephrine"); "1012388" (RxNorm: "epinephrine"); "1673242" (RxNorm: "epinephrine"); "1673243" (RxNorm: "epinephrine"); "1673296" (RxNorm: "epinephrine"); "1673298" (RxNorm: "epinephrine"); "17478020102" (NDC: "phenylephrine"); "65035902" (NDC: "phenylephrine"); "1372298" (RxNorm: "phenylephrine"); "63323048327" (NDC: "epinephrine"); "1150121" (RxNorm: "epinephrine"); "93598627" (NDC: "epinephrine"); "409175550" (NDC: "epinephrine"); "781346670" (NDC: "phenylephrine"); "409780924" (NDC: "dopamine"); "1549388" (RxNorm: "phenylephrine"); "63323048303" (NDC: "epinephrine"); "1090463" (RxNorm: "phenylephrine"); "1234571" (RxNorm: "phenylephrine"); "409234402" (NDC: "dobutamine"); "1293628" (RxNorm: "epinephrine"); "1608499" (RxNorm: "epinephrine"); "338100502" (NDC: "dopamine"); "17478020115" (NDC: "phenylephrine"); "63323048717" (NDC: "epinephrine"); "49502010201" (NDC: "epinephrine"); "55045320702" (NDC: "epinephrine"); "63323048357" (NDC: "epinephrine"); "49502050102" (NDC: "epinephrine"); "409372432" (NDC: "dobutamine"); "42367057022" (NDC: "vasopressin (USP)"); "60842002101" (NDC: "epinephrine"); "1991329" (RxNorm: "epinephrine"); "404651205" (NDC: "epinephrine"); "50580051501" (NDC: "phenylephrine"); "409318101" (NDC: "epinephrine"); "49502010101" (NDC: "epinephrine"); "49502010102" (NDC: "epinephrine"); "409234401" (NDC: "dobutamine"); "1087043" (RxNorm: "phenylephrine"); "1743869" (RxNorm: "dopamine"); "1743871" (RxNorm: "dopamine"); "63323048707" (NDC: "epinephrine"); "1086997" (RxNorm: "phenylephrine"); "1236048" (RxNorm: "phenylephrine"); "1305268" (RxNorm: "epinephrine"); "1870225" (RxNorm: "epinephrine"); "1991328" (RxNorm: "epinephrine"); "409492120" (NDC: "epinephrine"); "42023021301" (NDC: "phenylephrine"); "67457085200" (NDC: "norepinephrine"); "60842002301" (NDC: "epinephrine"); "1233546" (RxNorm: "phenylephrine"); "1870232" (RxNorm: "epinephrine"); "1673142" (RxNorm: "epinephrine"); |
| **Organ Dysfunction** | "J96.00" (ICD-10-CM: "Acute respiratory failure, unspecified whether with hypoxia or hypercapnia"); "N17.9" (ICD-10-CM: "Acute kidney failure, unspecified"); "J96.01" (ICD-10-CM: "Acute respiratory failure with hypoxia"); "J96.02" (ICD-10-CM: "Acute respiratory failure with hypercapnia"); "K72.00" (ICD-10-CM: "Acute and subacute hepatic failure without coma"); "N17.8" (ICD-10-CM: "Other acute kidney failure"); "N17.0" (ICD-10-CM: "Acute kidney failure with tubular necrosis"); "G72.81" (ICD-10-CM: "Critical illness myopathy"); "G93.41" (ICD-10-CM: "Metabolic encephalopathy"); "D65" (ICD-10-CM: "Disseminated intravascular coagulation [defibrination syndrome]"); "G62.81" (ICD-10-CM: "Critical illness polyneuropathy"); "K72.01" (ICD-10-CM: "Acute and subacute hepatic failure with coma"); "N17.1" (ICD-10-CM: "Acute kidney failure with acute cortical necrosis"); "J96.0" (ICD-10-CM: "Acute respiratory failure"); "N17" (ICD-10-CM: "Acute kidney failure"); "N17.2" (ICD-10-CM: "Acute kidney failure with medullary necrosis"); |
| **Mechanical Ventilation** | "0BH17EZ" (ICD-10-PCS: "Insertion of Endotracheal Airway into Trachea, Via Natural or Artificial Opening"); "0BH18EZ" (ICD-10-PCS: "Insertion of Endotracheal Airway into Trachea, Via Natural or Artificial Opening Endoscopic"); "31500" (CPT: "Intubation, endotracheal, emergency procedure"); "5A19054" (ICD-10-PCS: "Respiratory Ventilation, Single, Nonmechanical"); "5A1935Z" (ICD-10-PCS: "Respiratory Ventilation, Less than 24 Consecutive Hours"); "5A1945Z" (ICD-10-PCS: "Respiratory Ventilation, 24-96 Consecutive Hours"); "5A1955Z" (ICD-10-PCS: "Respiratory Ventilation, Greater than 96 Consecutive Hours"); "94002" (CPT: "Ventilation assist and management, initiation of pressure or volume preset ventilators for assisted or controlled breathing; hospital inpatient/observation, initial day"); "94003" (CPT: "Ventilation assist and management, initiation of pressure or volume preset ventilators for assisted or controlled breathing; hospital inpatient/observation, each subsequent day"); |
| **Respiratory Failure** | "J96.00" (ICD-10-CM: "Acute respiratory failure, unspecified whether with hypoxia or hypercapnia"); "J96.01" (ICD-10-CM: "Acute respiratory failure with hypoxia"); "J96.02" (ICD-10-CM: "Acute respiratory failure with hypercapnia"); "J80" (ICD-10-CM: "Acute respiratory distress syndrome"); "J96.20" (ICD-10-CM: "Acute and chronic respiratory failure, unspecified whether with hypoxia or hypercapnia"); "R09.2" (ICD-10-CM: "Respiratory arrest"); "J96.21" (ICD-10-CM: "Acute and chronic respiratory failure with hypoxia"); "J96.22" (ICD-10-CM: "Acute and chronic respiratory failure with hypercapnia"); "J96.0" (ICD-10-CM: "Acute respiratory failure"); "J96.2" (ICD-10-CM: "Acute and chronic respiratory failure"); |
| **Social Determinants of Health** | "Z59.1" (ICD-10-CM: "Inadequate housing"); "Z63.4" (ICD-10-CM: "Disappearance and death of family member"); "Z62.21" (ICD-10-CM: "Child in welfare custody"); "Z59.0" (ICD-10-CM: "Homelessness"); "Z59.00" (ICD-10-CM: "Homelessness unspecified"); "Z63.9" (ICD-10-CM: "Problem related to primary support group, unspecified"); "Z65.8" (ICD-10-CM: "Other specified problems related to psychosocial circumstances"); "Z60.8" (ICD-10-CM: "Other problems related to social environment"); "Z65.9" (ICD-10-CM: "Problem related to unspecified psychosocial circumstances"); "Z59.89" (ICD-10-CM: "Other problems related to housing and economic circumstances"); "Z55.9" (ICD-10-CM: "Problems related to education and literacy, unspecified"); "Z62.810" (ICD-10-CM: "Personal history of physical and sexual abuse in childhood"); "Z63.79" (ICD-10-CM: "Other stressful life events affecting family and household"); "Z62.820" (ICD-10-CM: "Parent-biological child conflict"); "Z63.0" (ICD-10-CM: "Problems in relationship with spouse or partner"); "Z63.8" (ICD-10-CM: "Other specified problems related to primary support group"); "Z60.4" (ICD-10-CM: "Social exclusion and rejection"); "Z62.891" (ICD-10-CM: "Sibling rivalry"); "Z63.5" (ICD-10-CM: "Disruption of family by separation and divorce"); "Z62.819" (ICD-10-CM: "Personal history of unspecified abuse in childhood"); "Z60.9" (ICD-10-CM: "Problem related to social environment, unspecified"); "Z62.898" (ICD-10-CM: "Other specified problems related to upbringing"); "Z55.8" (ICD-10-CM: "Other problems related to education and literacy"); "Z56.0" (ICD-10-CM: "Unemployment, unspecified"); "Z64.1" (ICD-10-CM: "Problems related to multiparity"); "Z65.1" (ICD-10-CM: "Imprisonment and other incarceration"); "Z59.9" (ICD-10-CM: "Problem related to housing and economic circumstances, unspecified"); "Z59.01" (ICD-10-CM: "Sheltered homelessness"); "Z59.86" (ICD-10-CM: "nan"); "Z65.3" (ICD-10-CM: "Problems related to other legal circumstances"); "Z60.2" (ICD-10-CM: "Problems related to living alone"); "Z59.02" (ICD-10-CM: "Unsheltered homelessness"); "Z57.31" (ICD-10-CM: "Occupational exposure to environmental tobacco smoke"); "Z59.3" (ICD-10-CM: "Problems related to living in residential institution"); "Z59.6" (ICD-10-CM: "Low income"); "Z59.7" (ICD-10-CM: "Insufficient social insurance and welfare support"); "Z55.3" (ICD-10-CM: "Underachievement in school"); "Z59.4" (ICD-10-CM: "Lack of adequate food"); "Z59.8" (ICD-10-CM: "Other problems related to housing and economic circumstances"); "Z62.821" (ICD-10-CM: "Parent-adopted child conflict"); "Z60.3" (ICD-10-CM: "Acculturation difficulty"); "Z56.3" (ICD-10-CM: "Stressful work schedule"); "Z62.6" (ICD-10-CM: "Inappropriate (excessive) parental pressure"); "Z59.41" (ICD-10-CM: "Food insecurity"); "Z63.32" (ICD-10-CM: "Other absence of family member"); "Z62.812" (ICD-10-CM: "Personal history of neglect in childhood"); "Z62.811" (ICD-10-CM: "Personal history of psychological abuse in childhood"); "Z65.4" (ICD-10-CM: "Victim of crime and terrorism"); "Z63.72" (ICD-10-CM: "Alcoholism and drug addiction in family"); "Z59.87" (ICD-10-CM: "nan"); "Z64.0" (ICD-10-CM: "Problems related to unwanted pregnancy"); "Z55.4" (ICD-10-CM: "Educational maladjustment and discord with teachers and classmates"); "Z59.819" (ICD-10-CM: "Housing instability, housed unspecified"); "Z57.2" (ICD-10-CM: "Occupational exposure to dust"); "Z57.8" (ICD-10-CM: "Occupational exposure to other risk factors"); "Z64.4" (ICD-10-CM: "Discord with counselors"); "Z59.811" (ICD-10-CM: "Housing instability, housed, with risk of homelessness"); "Z62.22" (ICD-10-CM: "Institutional upbringing"); "Z63.6" (ICD-10-CM: "Dependent relative needing care at home"); "Z62.29" (ICD-10-CM: "Other upbringing away from parents"); "Z62.890" (ICD-10-CM: "Parent-child estrangement NEC"); "Z60.0" (ICD-10-CM: "Problems of adjustment to life-cycle transitions"); "Z62.813" (ICD-10-CM: "Personal history of forced labor or sexual exploitation in childhood"); "Z59.2" (ICD-10-CM: "Discord with neighbors, lodgers and landlord"); "Z56.89" (ICD-10-CM: "Other problems related to employment"); "Z56.9" (ICD-10-CM: "Unspecified problems related to employment"); "Z59.5" (ICD-10-CM: "Extreme poverty"); "Z59.82" (ICD-10-CM: "nan"); "Z56.81" (ICD-10-CM: "Sexual harassment on the job"); "Z57.1" (ICD-10-CM: "Occupational exposure to radiation"); "Z62.822" (ICD-10-CM: "Parent-foster child conflict"); "Z62.0" (ICD-10-CM: "Inadequate parental supervision and control"); "Z57.9" (ICD-10-CM: "Occupational exposure to unspecified risk factor"); "Z55.0" (ICD-10-CM: "Illiteracy and low-level literacy"); "Z65.0" (ICD-10-CM: "Conviction in civil and criminal proceedings without imprisonment"); "Z59.812" (ICD-10-CM: "Housing instability, housed, homelessness in past 12 months"); "Z56.6" (ICD-10-CM: "Other physical and mental strain related to work"); "Z55.2" (ICD-10-CM: "Failed school examinations"); "Z59.48" (ICD-10-CM: "Other specified lack of adequate food"); "Z60.5" (ICD-10-CM: "Target of (perceived) adverse discrimination and persecution"); "Z62.3" (ICD-10-CM: "Hostility towards and scapegoating of child"); "Z55.1" (ICD-10-CM: "Schooling unavailable and unattainable"); "Z62.9" (ICD-10-CM: "Problem related to upbringing, unspecified"); "Z65.2" (ICD-10-CM: "Problems related to release from prison"); "Z56.1" (ICD-10-CM: "Change of job"); "Z57.5" (ICD-10-CM: "Occupational exposure to toxic agents in other industries"); "Z65.5" (ICD-10-CM: "Exposure to disaster, war and other hostilities"); "Z55.5" (ICD-10-CM: "Less than a high school diploma"); "Z56.2" (ICD-10-CM: "Threat of job loss"); "Z56.4" (ICD-10-CM: "Discord with boss and workmates"); "Z63.1" (ICD-10-CM: "Problems in relationship with in-laws"); "Z63.31" (ICD-10-CM: "Absence of family member due to military deployment"); "Z57.39" (ICD-10-CM: "Occupational exposure to other air contaminants"); "Z62.1" (ICD-10-CM: "Parental overprotection"); |
| **Sepsis** | "A41.9" (ICD-10-CM: "Sepsis, unspecified organism"); "R65.21" (ICD-10-CM: "Severe sepsis with septic shock"); "J18.9" (ICD-10-CM: "Pneumonia, unspecified organism"); "A41.1" (ICD-10-CM: "Sepsis due to other specified staphylococcus"); "A41.51" (ICD-10-CM: "Sepsis due to Escherichia coli [E. coli]"); "A41.59" (ICD-10-CM: "Other Gram-negative sepsis"); "N39.0" (ICD-10-CM: "Urinary tract infection, site not specified"); "B95.62" (ICD-10-CM: "Methicillin resistant Staphylococcus aureus infection as the cause of diseases classified elsewhere"); "R65.20" (ICD-10-CM: "Severe sepsis without septic shock"); "B95.61" (ICD-10-CM: "Methicillin susceptible Staphylococcus aureus infection as the cause of diseases classified elsewhere"); "A41.89" (ICD-10-CM: "Other specified sepsis"); "A39.4" (ICD-10-CM: "Meningococcemia, unspecified"); "A40.0" (ICD-10-CM: "Sepsis due to streptococcus, group A"); "A41.81" (ICD-10-CM: "Sepsis due to Enterococcus"); "A40.3" (ICD-10-CM: "Sepsis due to Streptococcus pneumoniae"); "A41.02" (ICD-10-CM: "Sepsis due to Methicillin resistant Staphylococcus aureus"); "A40.8" (ICD-10-CM: "Other streptococcal sepsis"); "B95.4" (ICD-10-CM: "Other streptococcus as the cause of diseases classified elsewhere"); "I76" (ICD-10-CM: "Septic arterial embolism"); "A41.01" (ICD-10-CM: "Sepsis due to Methicillin susceptible Staphylococcus aureus"); "A41.2" (ICD-10-CM: "Sepsis due to unspecified staphylococcus"); "A41.50" (ICD-10-CM: "Gram-negative sepsis, unspecified"); "J44.0" (ICD-10-CM: "Chronic obstructive pulmonary disease with (acute) lower respiratory infection"); "A41.4" (ICD-10-CM: "Sepsis due to anaerobes"); "A41.3" (ICD-10-CM: "Sepsis due to Hemophilus influenzae"); "A04.7" (ICD-10-CM: "Enterocolitis due to Clostridium difficile"); "A41.52" (ICD-10-CM: "Sepsis due to Pseudomonas"); "A40.9" (ICD-10-CM: "Streptococcal sepsis, unspecified"); "A40.1" (ICD-10-CM: "Sepsis due to streptococcus, group B"); "A32.7" (ICD-10-CM: "Listerial sepsis"); "B37.7" (ICD-10-CM: "Candidal sepsis"); "A02.1" (ICD-10-CM: "Salmonella sepsis"); "A39.2" (ICD-10-CM: "Acute meningococcemia"); "A28.0" (ICD-10-CM: "Pasteurellosis"); "A41" (ICD-10-CM: "Other sepsis"); "A41.53" (ICD-10-CM: "Sepsis due to Serratia"); "A03.9" (ICD-10-CM: "Shigellosis, unspecified"); "B00.7" (ICD-10-CM: "Disseminated herpesviral disease"); "A39.3" (ICD-10-CM: "Chronic meningococcemia"); "A23.9" (ICD-10-CM: "Brucellosis, unspecified"); |
| **Substance Use Disorder** | "F12.10" (ICD-10-CM: "Cannabis abuse, uncomplicated"); "O99.322" (ICD-10-CM: "Drug use complicating pregnancy, second trimester"); "F12.20" (ICD-10-CM: "Cannabis dependence, uncomplicated"); "F12.120" (ICD-10-CM: "Cannabis abuse with intoxication, uncomplicated"); "F12.19" (ICD-10-CM: "Cannabis abuse with unspecified cannabis-induced disorder"); "F12.90" (ICD-10-CM: "Cannabis use, unspecified, uncomplicated"); "F19.10" (ICD-10-CM: "Other psychoactive substance abuse, uncomplicated"); "F12.288" (ICD-10-CM: "Cannabis dependence with other cannabis-induced disorder"); "F12.988" (ICD-10-CM: "Cannabis use, unspecified with other cannabis-induced disorder"); "F12.99" (ICD-10-CM: "Cannabis use, unspecified with unspecified cannabis-induced disorder"); "O99.320" (ICD-10-CM: "Drug use complicating pregnancy, unspecified trimester"); "O99.321" (ICD-10-CM: "Drug use complicating pregnancy, first trimester"); "T40.7X1A" (ICD-10-CM: "Poisoning by cannabis (derivatives), accidental (unintentional), initial encounter (deprecated 2021)"); "F13.19" (ICD-10-CM: "Sedative, hypnotic or anxiolytic abuse with unspecified sedative, hypnotic or anxiolytic-induced disorder"); "F19.129" (ICD-10-CM: "Other psychoactive substance abuse with intoxication, unspecified"); "F11.20" (ICD-10-CM: "Opioid dependence, uncomplicated"); "F11.90" (ICD-10-CM: "Opioid use, unspecified, uncomplicated"); "F16.90" (ICD-10-CM: "Hallucinogen use, unspecified, uncomplicated"); "F19.20" (ICD-10-CM: "Other psychoactive substance dependence, uncomplicated"); "F19.929" (ICD-10-CM: "Other psychoactive substance use, unspecified with intoxication, unspecified"); "T40.1X1A" (ICD-10-CM: "Poisoning by heroin, accidental (unintentional), initial encounter"); "Z71.6" (ICD-10-CM: "Tobacco abuse counseling"); "F12.929" (ICD-10-CM: "Cannabis use, unspecified with intoxication, unspecified"); "F19.90" (ICD-10-CM: "Other psychoactive substance use, unspecified, uncomplicated"); "F14.10" (ICD-10-CM: "Cocaine abuse, uncomplicated"); "F19.19" (ICD-10-CM: "Other psychoactive substance abuse with unspecified psychoactive substance-induced disorder"); "F12.29" (ICD-10-CM: "Cannabis dependence with unspecified cannabis-induced disorder"); "F19.931" (ICD-10-CM: "Other psychoactive substance use, unspecified with withdrawal delirium"); "T40.411A" (ICD-10-CM: "Poisoning by fentanyl or fentanyl analogs, accidental (unintentional), initial encounter"); "F11.10" (ICD-10-CM: "Opioid abuse, uncomplicated"); "F13.10" (ICD-10-CM: "Sedative, hypnotic or anxiolytic abuse, uncomplicated"); "F14.129" (ICD-10-CM: "Cocaine abuse with intoxication, unspecified"); "T40.2X1A" (ICD-10-CM: "Poisoning by other opioids, accidental (unintentional), initial encounter"); "T40.601A" (ICD-10-CM: "Poisoning by unspecified narcotics, accidental (unintentional), initial encounter"); "F12.188" (ICD-10-CM: "Cannabis abuse with other cannabis-induced disorder"); "T40.604A" (ICD-10-CM: "Poisoning by unspecified narcotics, undetermined, initial encounter"); "F14.90" (ICD-10-CM: "Cocaine use, unspecified, uncomplicated"); "F16.10" (ICD-10-CM: "Hallucinogen abuse, uncomplicated"); "T40.8X1A" (ICD-10-CM: "Poisoning by lysergide [LSD], accidental (unintentional), initial encounter"); "F14.99" (ICD-10-CM: "Cocaine use, unspecified with unspecified cocaine-induced disorder"); "F19.99" (ICD-10-CM: "Other psychoactive substance use, unspecified with unspecified psychoactive substance-induced disorder"); "F12.980" (ICD-10-CM: "Cannabis use, unspecified with anxiety disorder"); "T40.2X2A" (ICD-10-CM: "Poisoning by other opioids, intentional self-harm, initial encounter"); "F19.939" (ICD-10-CM: "Other psychoactive substance use, unspecified with withdrawal, unspecified"); "T40.602A" (ICD-10-CM: "Poisoning by unspecified narcotics, intentional self-harm, initial encounter"); "F15.129" (ICD-10-CM: "Other stimulant abuse with intoxication, unspecified"); "T40.721A" (ICD-10-CM: "Poisoning by synthetic cannabinoids, accidental (unintentional), initial encounter"); "F12.121" (ICD-10-CM: "Cannabis abuse with intoxication delirium"); "T40.711A" (ICD-10-CM: "Poisoning by cannabis, accidental (unintentional), initial encounter"); "F17.218" (ICD-10-CM: "Nicotine dependence, cigarettes, with other nicotine-induced disorders"); "F15.10" (ICD-10-CM: "Other stimulant abuse, uncomplicated"); "F11.23" (ICD-10-CM: "Opioid dependence with withdrawal"); "F19.980" (ICD-10-CM: "Other psychoactive substance use, unspecified with psychoactive substance-induced anxiety disorder"); "F15.20" (ICD-10-CM: "Other stimulant dependence, uncomplicated"); "F15.229" (ICD-10-CM: "Other stimulant dependence with intoxication, unspecified"); "F15.259" (ICD-10-CM: "Other stimulant dependence with stimulant-induced psychotic disorder, unspecified"); "F13.90" (ICD-10-CM: "Sedative, hypnotic, or anxiolytic use, unspecified, uncomplicated"); "F15.90" (ICD-10-CM: "Other stimulant use, unspecified, uncomplicated"); "O99.324" (ICD-10-CM: "Drug use complicating childbirth"); "F19.222" (ICD-10-CM: "Other psychoactive substance dependence with intoxication with perceptual disturbance"); "F19.94" (ICD-10-CM: "Other psychoactive substance use, unspecified with psychoactive substance-induced mood disorder"); "F15.929" (ICD-10-CM: "Other stimulant use, unspecified with intoxication, unspecified"); "T40.4X2A" (ICD-10-CM: "Poisoning by other synthetic narcotics, intentional self-harm, initial encounter"); "F19.921" (ICD-10-CM: "Other psychoactive substance use, unspecified with intoxication with delirium"); "F13.139" (ICD-10-CM: "Sedative, hypnotic or anxiolytic abuse with withdrawal, unspecified"); "F19.959" (ICD-10-CM: "Other psychoactive substance use, unspecified with psychoactive substance-induced psychotic disorder, unspecified"); "F17.299" (ICD-10-CM: "Nicotine dependence, other tobacco product, with unspecified nicotine-induced disorders"); "F15.959" (ICD-10-CM: "Other stimulant use, unspecified with stimulant-induced psychotic disorder, unspecified"); "F19.14" (ICD-10-CM: "Other psychoactive substance abuse with psychoactive substance-induced mood disorder"); "O35.5XX0" (ICD-10-CM: "Maternal care for (suspected) damage to fetus by drugs, not applicable or unspecified"); "O35.5XX1" (ICD-10-CM: "Maternal care for (suspected) damage to fetus by drugs, fetus 1"); "O99.323" (ICD-10-CM: "Drug use complicating pregnancy, third trimester"); "O99.325" (ICD-10-CM: "Drug use complicating the puerperium"); "Z71.51" (ICD-10-CM: "Drug abuse counseling and surveillance of drug abuser"); "F19.239" (ICD-10-CM: "Other psychoactive substance dependence with withdrawal, unspecified"); "F14.120" (ICD-10-CM: "Cocaine abuse with intoxication, uncomplicated"); "F14.929" (ICD-10-CM: "Cocaine use, unspecified with intoxication, unspecified"); "F16.959" (ICD-10-CM: "Hallucinogen use, unspecified with hallucinogen-induced psychotic disorder, unspecified"); "Z71.41" (ICD-10-CM: "Alcohol abuse counseling and surveillance of alcoholic"); "T40.1X2A" (ICD-10-CM: "Poisoning by heroin, intentional self-harm, initial encounter"); "T40.492A" (ICD-10-CM: "Poisoning by other synthetic narcotics, intentional self-harm, initial encounter"); "F13.20" (ICD-10-CM: "Sedative, hypnotic or anxiolytic dependence, uncomplicated"); "T40.4X1A" (ICD-10-CM: "Poisoning by other synthetic narcotics, accidental (unintentional), initial encounter"); "T40.691A" (ICD-10-CM: "Poisoning by other narcotics, accidental (unintentional), initial encounter"); "F12.129" (ICD-10-CM: "Cannabis abuse with intoxication, unspecified"); "F13.239" (ICD-10-CM: "Sedative, hypnotic or anxiolytic dependence with withdrawal, unspecified"); "F11.188" (ICD-10-CM: "Opioid abuse with other opioid-induced disorder"); "F11.129" (ICD-10-CM: "Opioid abuse with intoxication, unspecified"); "F11.19" (ICD-10-CM: "Opioid abuse with unspecified opioid-induced disorder"); "F11.229" (ICD-10-CM: "Opioid dependence with intoxication, unspecified"); "T40.3X1A" (ICD-10-CM: "Poisoning by methadone, accidental (unintentional), initial encounter"); "F12.950" (ICD-10-CM: "Cannabis use, unspecified with psychotic disorder with delusions"); "F12.951" (ICD-10-CM: "Cannabis use, unspecified with psychotic disorder with hallucinations"); "F15.151" (ICD-10-CM: "Other stimulant abuse with stimulant-induced psychotic disorder with hallucinations"); "F17.228" (ICD-10-CM: "Nicotine dependence, chewing tobacco, with other nicotine-induced disorders"); "F15.23" (ICD-10-CM: "Other stimulant dependence with withdrawal"); "F55.8" (ICD-10-CM: "Abuse of other non-psychoactive substances"); "F13.231" (ICD-10-CM: "Sedative, hypnotic or anxiolytic dependence with withdrawal delirium"); "F12.159" (ICD-10-CM: "Cannabis abuse with psychotic disorder, unspecified"); "F15.159" (ICD-10-CM: "Other stimulant abuse with stimulant-induced psychotic disorder, unspecified"); "T40.991A" (ICD-10-CM: "Poisoning by other psychodysleptics [hallucinogens], accidental (unintentional), initial encounter"); "F12.921" (ICD-10-CM: "Cannabis use, unspecified with intoxication delirium"); "F15.14" (ICD-10-CM: "Other stimulant abuse with stimulant-induced mood disorder"); "F16.151" (ICD-10-CM: "Hallucinogen abuse with hallucinogen-induced psychotic disorder with hallucinations"); "F19.121" (ICD-10-CM: "Other psychoactive substance abuse with intoxication delirium"); "T40.2X4A" (ICD-10-CM: "Poisoning by other opioids, undetermined, initial encounter"); "F55.2" (ICD-10-CM: "Abuse of laxatives"); "F11.29" (ICD-10-CM: "Opioid dependence with unspecified opioid-induced disorder"); "F16.99" (ICD-10-CM: "Hallucinogen use, unspecified with unspecified hallucinogen-induced disorder"); "F18.10" (ICD-10-CM: "Inhalant abuse, uncomplicated"); "F15.121" (ICD-10-CM: "Other stimulant abuse with intoxication delirium"); "F13.129" (ICD-10-CM: "Sedative, hypnotic or anxiolytic abuse with intoxication, unspecified"); "T40.3X2A" (ICD-10-CM: "Poisoning by methadone, intentional self-harm, initial encounter"); "F14.151" (ICD-10-CM: "Cocaine abuse with cocaine-induced psychotic disorder with hallucinations"); "F19.151" (ICD-10-CM: "Other psychoactive substance abuse with psychoactive substance-induced psychotic disorder with hallucinations"); "F11.24" (ICD-10-CM: "Opioid dependence with opioid-induced mood disorder"); "F11.288" (ICD-10-CM: "Opioid dependence with other opioid-induced disorder"); "F19.951" (ICD-10-CM: "Other psychoactive substance use, unspecified with psychoactive substance-induced psychotic disorder with hallucinations"); "F11.93" (ICD-10-CM: "Opioid use, unspecified with withdrawal"); "F11.99" (ICD-10-CM: "Opioid use, unspecified with unspecified opioid-induced disorder"); "F15.99" (ICD-10-CM: "Other stimulant use, unspecified with unspecified stimulant-induced disorder"); "F12.13" (ICD-10-CM: "Cannabis abuse with withdrawal"); "F14.20" (ICD-10-CM: "Cocaine dependence, uncomplicated"); "F13.920" (ICD-10-CM: "Sedative, hypnotic or anxiolytic use, unspecified with intoxication, uncomplicated"); "F14.920" (ICD-10-CM: "Cocaine use, unspecified with intoxication, uncomplicated"); "F19.120" (ICD-10-CM: "Other psychoactive substance abuse with intoxication, uncomplicated"); "F14.19" (ICD-10-CM: "Cocaine abuse with unspecified cocaine-induced disorder"); "F12.920" (ICD-10-CM: "Cannabis use, unspecified with intoxication, uncomplicated"); "F19.29" (ICD-10-CM: "Other psychoactive substance dependence with unspecified psychoactive substance-induced disorder"); "F14.188" (ICD-10-CM: "Cocaine abuse with other cocaine-induced disorder"); "F12.221" (ICD-10-CM: "Cannabis dependence with intoxication delirium"); "T40.425A" (ICD-10-CM: "Adverse effect of tramadol, initial encounter"); "F19.920" (ICD-10-CM: "Other psychoactive substance use, unspecified with intoxication, uncomplicated"); "T40.414A" (ICD-10-CM: "Poisoning by fentanyl or fentanyl analogs, undetermined, initial encounter"); "F11.121" (ICD-10-CM: "Opioid abuse with intoxication delirium"); "F11.13" (ICD-10-CM: "Opioid abuse with withdrawal"); "F11.920" (ICD-10-CM: "Opioid use, unspecified with intoxication, uncomplicated"); "F15.120" (ICD-10-CM: "Other stimulant abuse with intoxication, uncomplicated"); "F11.929" (ICD-10-CM: "Opioid use, unspecified with intoxication, unspecified"); "F17.213" (ICD-10-CM: "Nicotine dependence, cigarettes, with withdrawal"); "F19.950" (ICD-10-CM: "Other psychoactive substance use, unspecified with psychoactive substance-induced psychotic disorder with delusions"); "F16.920" (ICD-10-CM: "Hallucinogen use, unspecified with intoxication, uncomplicated"); "T40.412A" (ICD-10-CM: "Poisoning by fentanyl or fentanyl analogs, intentional self-harm, initial encounter"); "F19.131" (ICD-10-CM: "Other psychoactive substance abuse with withdrawal delirium"); "F19.139" (ICD-10-CM: "Other psychoactive substance abuse with withdrawal, unspecified"); "F16.121" (ICD-10-CM: "Hallucinogen abuse with intoxication with delirium"); "T40.3X4A" (ICD-10-CM: "Poisoning by methadone, undetermined, initial encounter"); "F17.209" (ICD-10-CM: "Nicotine dependence, unspecified, with unspecified nicotine-induced disorders"); "F11.120" (ICD-10-CM: "Opioid abuse with intoxication, uncomplicated"); "F15.920" (ICD-10-CM: "Other stimulant use, unspecified with intoxication, uncomplicated"); "F13.230" (ICD-10-CM: "Sedative, hypnotic or anxiolytic dependence with withdrawal, uncomplicated"); "F13.930" (ICD-10-CM: "Sedative, hypnotic or anxiolytic use, unspecified with withdrawal, uncomplicated"); "F13.939" (ICD-10-CM: "Sedative, hypnotic or anxiolytic use, unspecified with withdrawal, unspecified"); "F17.298" (ICD-10-CM: "Nicotine dependence, other tobacco product, with other nicotine-induced disorders"); "F15.93" (ICD-10-CM: "Other stimulant use, unspecified with withdrawal"); "F13.29" (ICD-10-CM: "Sedative, hypnotic or anxiolytic dependence with unspecified sedative, hypnotic or anxiolytic-induced disorder"); "F13.931" (ICD-10-CM: "Sedative, hypnotic or anxiolytic use, unspecified with withdrawal delirium"); "T40.0X2A" (ICD-10-CM: "Poisoning by opium, intentional self-harm, initial encounter"); "F13.121" (ICD-10-CM: "Sedative, hypnotic or anxiolytic abuse with intoxication delirium"); "F16.122" (ICD-10-CM: "Hallucinogen abuse with intoxication with perceptual disturbance"); "F11.221" (ICD-10-CM: "Opioid dependence with intoxication delirium"); "F15.221" (ICD-10-CM: "Other stimulant dependence with intoxication delirium"); "F13.99" (ICD-10-CM: "Sedative, hypnotic or anxiolytic use, unspecified with unspecified sedative, hypnotic or anxiolytic-induced disorder"); "F16.120" (ICD-10-CM: "Hallucinogen abuse with intoxication, uncomplicated"); "F19.922" (ICD-10-CM: "Other psychoactive substance use, unspecified with intoxication with perceptual disturbance"); "F15.951" (ICD-10-CM: "Other stimulant use, unspecified with stimulant-induced psychotic disorder with hallucinations"); "F12.959" (ICD-10-CM: "Cannabis use, unspecified with psychotic disorder, unspecified"); "F14.14" (ICD-10-CM: "Cocaine abuse with cocaine-induced mood disorder"); "F12.259" (ICD-10-CM: "Cannabis dependence with psychotic disorder, unspecified"); "F16.20" (ICD-10-CM: "Hallucinogen dependence, uncomplicated"); "F15.24" (ICD-10-CM: "Other stimulant dependence with stimulant-induced mood disorder"); "F13.988" (ICD-10-CM: "Sedative, hypnotic or anxiolytic use, unspecified with other sedative, hypnotic or anxiolytic-induced disorder"); "F19.159" (ICD-10-CM: "Other psychoactive substance abuse with psychoactive substance-induced psychotic disorder, unspecified"); "F12.151" (ICD-10-CM: "Cannabis abuse with psychotic disorder with hallucinations"); "T40.0X1A" (ICD-10-CM: "Poisoning by opium, accidental (unintentional), initial encounter"); "F19.24" (ICD-10-CM: "Other psychoactive substance dependence with psychoactive substance-induced mood disorder"); "F16.929" (ICD-10-CM: "Hallucinogen use, unspecified with intoxication, unspecified"); "F19.231" (ICD-10-CM: "Other psychoactive substance dependence with withdrawal delirium"); "F17.219" (ICD-10-CM: "Nicotine dependence, cigarettes, with unspecified nicotine-induced disorders"); "F14.23" (ICD-10-CM: "Cocaine dependence with withdrawal"); "F19.988" (ICD-10-CM: "Other psychoactive substance use, unspecified with other psychoactive substance-induced disorder"); "T40.1X4A" (ICD-10-CM: "Poisoning by heroin, undetermined, initial encounter"); "F18.90" (ICD-10-CM: "Inhalant use, unspecified, uncomplicated"); "F15.988" (ICD-10-CM: "Other stimulant use, unspecified with other stimulant-induced disorder"); "F16.921" (ICD-10-CM: "Hallucinogen use, unspecified with intoxication with delirium"); "F19.982" (ICD-10-CM: "Other psychoactive substance use, unspecified with psychoactive substance-induced sleep disorder"); "Z71.52" (ICD-10-CM: "Counseling for family member of drug abuser"); "F17.203" (ICD-10-CM: "Nicotine dependence unspecified, with withdrawal"); "F17.293" (ICD-10-CM: "Nicotine dependence, other tobacco product, with withdrawal"); "F14.24" (ICD-10-CM: "Cocaine dependence with cocaine-induced mood disorder"); "F13.24" (ICD-10-CM: "Sedative, hypnotic or anxiolytic dependence with sedative, hypnotic or anxiolytic-induced mood disorder"); "T40.0X5A" (ICD-10-CM: "Adverse effect of opium, initial encounter"); "F12.229" (ICD-10-CM: "Cannabis dependence with intoxication, unspecified"); "F13.120" (ICD-10-CM: "Sedative, hypnotic or anxiolytic abuse with intoxication, uncomplicated"); "F14.13" (ICD-10-CM: "Cocaine abuse, unspecified with withdrawal"); "F11.921" (ICD-10-CM: "Opioid use, unspecified with intoxication delirium"); "F13.232" (ICD-10-CM: "Sedative, hypnotic or anxiolytic dependence with withdrawal with perceptual disturbance"); "F13.94" (ICD-10-CM: "Sedative, hypnotic or anxiolytic use, unspecified with sedative, hypnotic or anxiolytic-induced mood disorder"); "F11.14" (ICD-10-CM: "Opioid abuse with opioid-induced mood disorder"); "F16.950" (ICD-10-CM: "Hallucinogen use, unspecified with hallucinogen-induced psychotic disorder with delusions"); "F14.922" (ICD-10-CM: "Cocaine use, unspecified with intoxication with perceptual disturbance"); "F16.129" (ICD-10-CM: "Hallucinogen abuse with intoxication, unspecified"); "F13.921" (ICD-10-CM: "Sedative, hypnotic or anxiolytic use, unspecified with intoxication delirium"); "T40.422A" (ICD-10-CM: "Poisoning by tramadol, intentional self-harm, initial encounter"); "F19.150" (ICD-10-CM: "Other psychoactive substance abuse with psychoactive substance-induced psychotic disorder with delusions"); "F11.950" (ICD-10-CM: "Opioid use, unspecified with opioid-induced psychotic disorder with delusions"); "F12.150" (ICD-10-CM: "Cannabis abuse with psychotic disorder with delusions"); "F14.950" (ICD-10-CM: "Cocaine use, unspecified with cocaine-induced psychotic disorder with delusions"); "F14.959" (ICD-10-CM: "Cocaine use, unspecified with cocaine-induced psychotic disorder, unspecified"); "F15.150" (ICD-10-CM: "Other stimulant abuse with stimulant-induced psychotic disorder with delusions"); "F19.17" (ICD-10-CM: "Other psychoactive substance abuse with psychoactive substance-induced persisting dementia"); "F13.229" (ICD-10-CM: "Sedative, hypnotic or anxiolytic dependence with intoxication, unspecified"); "F16.229" (ICD-10-CM: "Hallucinogen dependence with intoxication, unspecified"); "F14.921" (ICD-10-CM: "Cocaine use, unspecified with intoxication delirium"); "F15.94" (ICD-10-CM: "Other stimulant use, unspecified with stimulant-induced mood disorder"); "F16.983" (ICD-10-CM: "Hallucinogen use, unspecified with hallucinogen persisting perception disorder (flashbacks)"); "F13.932" (ICD-10-CM: "Sedative, hypnotic or anxiolytic use, unspecified with withdrawal with perceptual disturbances"); "F15.921" (ICD-10-CM: "Other stimulant use, unspecified with intoxication delirium"); "T40.4X4A" (ICD-10-CM: "Poisoning by other synthetic narcotics, undetermined, initial encounter"); "F13.929" (ICD-10-CM: "Sedative, hypnotic or anxiolytic use, unspecified with intoxication, unspecified"); "T40.415A" (ICD-10-CM: "Adverse effect of fentanyl or fentanyl analogs, initial encounter"); "F55.3" (ICD-10-CM: "Abuse of steroids or hormones"); "F16.951" (ICD-10-CM: "Hallucinogen use, unspecified with hallucinogen-induced psychotic disorder with hallucinations"); "F12.180" (ICD-10-CM: "Cannabis abuse with cannabis-induced anxiety disorder"); "F13.151" (ICD-10-CM: "Sedative, hypnotic or anxiolytic abuse with sedative, hypnotic or anxiolytic-induced psychotic disorder with hallucinations"); "F14.150" (ICD-10-CM: "Cocaine abuse with cocaine-induced psychotic disorder with delusions"); "F15.250" (ICD-10-CM: "Other stimulant dependence with stimulant-induced psychotic disorder with delusions"); "T40.3X5A" (ICD-10-CM: "Adverse effect of methadone, initial encounter"); "F12.251" (ICD-10-CM: "Cannabis dependence with psychotic disorder with hallucinations"); "F18.17" (ICD-10-CM: "Inhalant abuse with inhalant-induced dementia"); "F19.280" (ICD-10-CM: "Other psychoactive substance dependence with psychoactive substance-induced anxiety disorder"); "F19.229" (ICD-10-CM: "Other psychoactive substance dependence with intoxication, unspecified"); "F11.988" (ICD-10-CM: "Opioid use, unspecified with other opioid-induced disorder"); "F14.229" (ICD-10-CM: "Cocaine dependence with intoxication, unspecified"); "T40.692A" (ICD-10-CM: "Poisoning by other narcotics, intentional self-harm, initial encounter"); "F12.250" (ICD-10-CM: "Cannabis dependence with psychotic disorder with delusions"); "F14.94" (ICD-10-CM: "Cocaine use, unspecified with cocaine-induced mood disorder"); "F19.259" (ICD-10-CM: "Other psychoactive substance dependence with psychoactive substance-induced psychotic disorder, unspecified"); "F15.922" (ICD-10-CM: "Other stimulant use, unspecified with intoxication with perceptual disturbance"); "F12.220" (ICD-10-CM: "Cannabis dependence with intoxication, uncomplicated"); "F14.220" (ICD-10-CM: "Cocaine dependence with intoxication, uncomplicated"); "F15.19" (ICD-10-CM: "Other stimulant abuse with unspecified stimulant-induced disorder"); "F14.93" (ICD-10-CM: "Cocaine use, unspecified with withdrawal"); "F15.188" (ICD-10-CM: "Other stimulant abuse with other stimulant-induced disorder"); "F19.188" (ICD-10-CM: "Other psychoactive substance abuse with other psychoactive substance-induced disorder"); "F13.14" (ICD-10-CM: "Sedative, hypnotic or anxiolytic abuse with sedative, hypnotic or anxiolytic-induced mood disorder"); "F15.13" (ICD-10-CM: "Other stimulant abuse with withdrawal"); "F12.222" (ICD-10-CM: "Cannabis dependence with intoxication with perceptual disturbance"); "F12.922" (ICD-10-CM: "Cannabis use, unspecified with intoxication with perceptual disturbance"); "F14.122" (ICD-10-CM: "Cocaine abuse with intoxication with perceptual disturbance"); "F55.1" (ICD-10-CM: "Abuse of herbal or folk remedies"); "F14.121" (ICD-10-CM: "Cocaine abuse with intoxication with delirium"); "F19.230" (ICD-10-CM: "Other psychoactive substance dependence with withdrawal, uncomplicated"); "F19.251" (ICD-10-CM: "Other psychoactive substance dependence with psychoactive substance-induced psychotic disorder with hallucinations"); "F18.929" (ICD-10-CM: "Inhalant use, unspecified with intoxication, unspecified"); "F18.980" (ICD-10-CM: "Inhalant use, unspecified with inhalant-induced anxiety disorder"); "F11.94" (ICD-10-CM: "Opioid use, unspecified with opioid-induced mood disorder"); "F15.950" (ICD-10-CM: "Other stimulant use, unspecified with stimulant-induced psychotic disorder with delusions"); "T40.491A" (ICD-10-CM: "Poisoning by other synthetic narcotics, accidental (unintentional), initial encounter"); "F13.188" (ICD-10-CM: "Sedative, hypnotic or anxiolytic abuse with other sedative, hypnotic or anxiolytic-induced disorder"); "F16.188" (ICD-10-CM: "Hallucinogen abuse with other hallucinogen-induced disorder"); "F14.980" (ICD-10-CM: "Cocaine use, unspecified with cocaine-induced anxiety disorder"); "F15.980" (ICD-10-CM: "Other stimulant use, unspecified with stimulant-induced anxiety disorder"); "F19.288" (ICD-10-CM: "Other psychoactive substance dependence with other psychoactive substance-induced disorder"); "F15.220" (ICD-10-CM: "Other stimulant dependence with intoxication, uncomplicated"); "T40.901A" (ICD-10-CM: "Poisoning by unspecified psychodysleptics [hallucinogens], accidental (unintentional), initial encounter"); "F15.29" (ICD-10-CM: "Other stimulant dependence with unspecified stimulant-induced disorder"); "F55.4" (ICD-10-CM: "Abuse of vitamins"); "F18.988" (ICD-10-CM: "Inhalant use, unspecified with other inhalant-induced disorder"); "F19.122" (ICD-10-CM: "Other psychoactive substance abuse with intoxication with perceptual disturbances"); "F19.232" (ICD-10-CM: "Other psychoactive substance dependence with withdrawal with perceptual disturbance"); "F14.159" (ICD-10-CM: "Cocaine abuse with cocaine-induced psychotic disorder, unspecified"); "F14.180" (ICD-10-CM: "Cocaine abuse with cocaine-induced anxiety disorder"); "F16.19" (ICD-10-CM: "Hallucinogen abuse with unspecified hallucinogen-induced disorder"); "F13.251" (ICD-10-CM: "Sedative, hypnotic or anxiolytic dependence with sedative, hypnotic or anxiolytic-induced psychotic disorder with hallucinations"); "F16.159" (ICD-10-CM: "Hallucinogen abuse with hallucinogen-induced psychotic disorder, unspecified"); "F11.959" (ICD-10-CM: "Opioid use, unspecified with opioid-induced psychotic disorder, unspecified"); "F18.20" (ICD-10-CM: "Inhalant dependence, uncomplicated"); "F18.180" (ICD-10-CM: "Inhalant abuse with inhalant-induced anxiety disorder"); "F18.188" (ICD-10-CM: "Inhalant abuse with other inhalant-induced disorder"); "F12.280" (ICD-10-CM: "Cannabis dependence with cannabis-induced anxiety disorder"); "F15.280" (ICD-10-CM: "Other stimulant dependence with stimulant-induced anxiety disorder"); "F15.180" (ICD-10-CM: "Other stimulant abuse with stimulant-induced anxiety disorder"); "F13.130" (ICD-10-CM: "Sedative, hypnotic or anxiolytic abuse with withdrawal, uncomplicated"); "F19.221" (ICD-10-CM: "Other psychoactive substance dependence with intoxication delirium"); "F18.19" (ICD-10-CM: "Inhalant abuse with unspecified inhalant-induced disorder"); "F13.159" (ICD-10-CM: "Sedative, hypnotic or anxiolytic abuse with sedative, hypnotic or anxiolytic-induced psychotic disorder, unspecified"); "T40.494A" (ICD-10-CM: "Poisoning by other synthetic narcotics, undetermined, initial encounter"); "F15.122" (ICD-10-CM: "Other stimulant abuse with intoxication with perceptual disturbance"); "F11.981" (ICD-10-CM: "Opioid use, unspecified with opioid-induced sexual dysfunction"); "F17.208" (ICD-10-CM: "Nicotine dependence, unspecified, with other nicotine-induced disorders"); "F13.220" (ICD-10-CM: "Sedative, hypnotic or anxiolytic dependence with intoxication, uncomplicated"); "F11.222" (ICD-10-CM: "Opioid dependence with intoxication with perceptual disturbance"); "F13.951" (ICD-10-CM: "Sedative, hypnotic or anxiolytic use, unspecified with sedative, hypnotic or anxiolytic-induced psychotic disorder with hallucinations"); "F16.288" (ICD-10-CM: "Hallucinogen dependence with other hallucinogen-induced disorder"); "F12.122" (ICD-10-CM: "Cannabis abuse with intoxication with perceptual disturbance"); "T40.0X4A" (ICD-10-CM: "Poisoning by opium, undetermined, initial encounter"); "F13.97" (ICD-10-CM: "Sedative, hypnotic or anxiolytic use, unspecified with sedative, hypnotic or anxiolytic-induced persisting dementia"); "F16.150" (ICD-10-CM: "Hallucinogen abuse with hallucinogen-induced psychotic disorder with delusions"); "F16.14" (ICD-10-CM: "Hallucinogen abuse with hallucinogen-induced mood disorder"); "F16.180" (ICD-10-CM: "Hallucinogen abuse with hallucinogen-induced anxiety disorder"); "F16.94" (ICD-10-CM: "Hallucinogen use, unspecified with hallucinogen-induced mood disorder"); "F16.988" (ICD-10-CM: "Hallucinogen use, unspecified with other hallucinogen-induced disorder"); "F14.988" (ICD-10-CM: "Cocaine use, unspecified with other cocaine-induced disorder"); "F11.159" (ICD-10-CM: "Opioid abuse with opioid-induced psychotic disorder, unspecified"); "T40.694A" (ICD-10-CM: "Poisoning by other narcotics, undetermined, initial encounter"); "F19.180" (ICD-10-CM: "Other psychoactive substance abuse with psychoactive substance-induced anxiety disorder"); "F13.288" (ICD-10-CM: "Sedative, hypnotic or anxiolytic dependence with other sedative, hypnotic or anxiolytic-induced disorder"); "F18.959" (ICD-10-CM: "Inhalant use, unspecified with inhalant-induced psychotic disorder, unspecified"); "T40.424A" (ICD-10-CM: "Poisoning by tramadol, undetermined, initial encounter"); "F14.29" (ICD-10-CM: "Cocaine dependence with unspecified cocaine-induced disorder"); "F15.982" (ICD-10-CM: "Other stimulant use, unspecified with stimulant-induced sleep disorder"); "F16.183" (ICD-10-CM: "Hallucinogen abuse with hallucinogen persisting perception disorder (flashbacks)"); "305.20" (ICD-9-CM: "Cannabis abuse, unspecified"); "292.9" (ICD-9-CM: "Unspecified drug-induced mental disorder"); "V65.42" (ICD-9-CM: "Counseling on substance use and abuse"); "304.00" (ICD-9-CM: "Opioid type dependence, unspecified"); "292.81" (ICD-9-CM: "Drug-induced delirium"); "965.00" (ICD-9-CM: "Poisoning by opium (alkaloids), unspecified"); "E850.2" (ICD-9-CM: "Accidental poisoning by other opiates and related narcotics"); "305.90" (ICD-9-CM: "Other, mixed, or unspecified drug abuse, unspecified"); "305.40" (ICD-9-CM: "Sedative, hypnotic or anxiolytic abuse, unspecified"); "304.70" (ICD-9-CM: "Combinations of opioid type drug with any other drug dependence, unspecified"); "305.22" (ICD-9-CM: "Cannabis abuse, episodic"); "305.50" (ICD-9-CM: "Opioid abuse, unspecified"); "305.51" (ICD-9-CM: "Opioid abuse, continuous"); "965.01" (ICD-9-CM: "Poisoning by heroin"); "965.09" (ICD-9-CM: "Poisoning by other opiates and related narcotics"); "E850.0" (ICD-9-CM: "Accidental poisoning by heroin"); "292.89" (ICD-9-CM: "Other specified drug-induced mental disorders"); "292.84" (ICD-9-CM: "Drug-induced mood disorder"); "304.30" (ICD-9-CM: "Cannabis dependence, unspecified"); "305.60" (ICD-9-CM: "Cocaine abuse, unspecified"); "305.21" (ICD-9-CM: "Cannabis abuse, continuous"); "305.91" (ICD-9-CM: "Other, mixed, or unspecified drug abuse, continuous"); "305.70" (ICD-9-CM: "Amphetamine or related acting sympathomimetic abuse, unspecified"); "305.71" (ICD-9-CM: "Amphetamine or related acting sympathomimetic abuse, continuous"); "304.31" (ICD-9-CM: "Cannabis dependence, continuous"); "304.41" (ICD-9-CM: "Amphetamine and other psychostimulant dependence, continuous"); "304.01" (ICD-9-CM: "Opioid type dependence, continuous"); "305.52" (ICD-9-CM: "Opioid abuse, episodic"); "304.80" (ICD-9-CM: "Combinations of drug dependence excluding opioid type drug, unspecified"); "304.81" (ICD-9-CM: "Combinations of drug dependence excluding opioid type drug, continuous"); "305.30" (ICD-9-CM: "Hallucinogen abuse, unspecified"); "304.61" (ICD-9-CM: "Other specified drug dependence, continuous"); "304.40" (ICD-9-CM: "Amphetamine and other psychostimulant dependence, unspecified"); "292.0" (ICD-9-CM: "Drug withdrawal"); "304.90" (ICD-9-CM: "Unspecified drug dependence, unspecified"); "965.02" (ICD-9-CM: "Poisoning by methadone"); "E850.1" (ICD-9-CM: "Accidental poisoning by methadone"); "304.72" (ICD-9-CM: "Combinations of opioid type drug with any other drug dependence, episodic"); "292.2" (ICD-9-CM: "Pathological drug intoxication"); "304.02" (ICD-9-CM: "Opioid type dependence, episodic"); "305.41" (ICD-9-CM: "Sedative, hypnotic or anxiolytic abuse, continuous"); "304.71" (ICD-9-CM: "Combinations of opioid type drug with any other drug dependence, continuous"); "292.85" (ICD-9-CM: "Drug induced sleep disorders"); "304.12" (ICD-9-CM: "Sedative, hypnotic or anxiolytic dependence, episodic"); "304.10" (ICD-9-CM: "Sedative, hypnotic or anxiolytic dependence, unspecified"); "304.20" (ICD-9-CM: "Cocaine dependence, unspecified"); "305.92" (ICD-9-CM: "Other, mixed, or unspecified drug abuse, episodic"); "304.50" (ICD-9-CM: "Hallucinogen dependence, unspecified"); "E854.1" (ICD-9-CM: "Accidental poisoning by psychodysleptics [hallucinogens]"); "292.12" (ICD-9-CM: "Drug-induced psychotic disorder with hallucinations"); "292.11" (ICD-9-CM: "Drug-induced psychotic disorder with delusions"); "304.91" (ICD-9-CM: "Unspecified drug dependence, continuous"); "305.62" (ICD-9-CM: "Cocaine abuse, episodic"); "304.32" (ICD-9-CM: "Cannabis dependence, episodic"); "648.31" (ICD-9-CM: "Drug dependence of mother, delivered, with or without mention of antepartum condition"); "648.33" (ICD-9-CM: "Drug dependence of mother, antepartum condition or complication"); "655.53" (ICD-9-CM: "Suspected damage to fetus from drugs, affecting management of mother, antepartum condition or complication"); "304.60" (ICD-9-CM: "Other specified drug dependence, unspecified"); "304.82" (ICD-9-CM: "Combinations of drug dependence excluding opioid type drug, episodic"); "304.21" (ICD-9-CM: "Cocaine dependence, continuous"); "760.75" (ICD-9-CM: "Cocaine affecting fetus or newborn via placenta or breast milk"); "305.61" (ICD-9-CM: "Cocaine abuse, continuous"); "305.81" (ICD-9-CM: "Antidepressant type abuse, continuous"); "305.7" (ICD-9-CM: "Amphetamine or related acting sympathomimetic abuse"); "304.11" (ICD-9-CM: "Sedative, hypnotic or anxiolytic dependence, continuous"); "305.32" (ICD-9-CM: "Hallucinogen abuse, episodic"); "304.3" (ICD-9-CM: "Cannabis dependence"); "305.82" (ICD-9-CM: "Antidepressant type abuse, episodic"); "304.51" (ICD-9-CM: "Hallucinogen dependence, continuous"); "305.42" (ICD-9-CM: "Sedative, hypnotic or anxiolytic abuse, episodic"); "305.31" (ICD-9-CM: "Hallucinogen abuse, continuous"); "760.72" (ICD-9-CM: "Narcotics affecting fetus or newborn via placenta or breast milk"); "305.72" (ICD-9-CM: "Amphetamine or related acting sympathomimetic abuse, episodic"); "304.42" (ICD-9-CM: "Amphetamine and other psychostimulant dependence, episodic"); "304.92" (ICD-9-CM: "Unspecified drug dependence, episodic"); "305.80" (ICD-9-CM: "Antidepressant type abuse, unspecified"); "E935.1" (ICD-9-CM: "Methadone causing averse effects in therapeutic use"); "648.30" (ICD-9-CM: "Drug dependence of mother, unspecified as to episode of care or not applicable"); "304.52" (ICD-9-CM: "Hallucinogen dependence, episodic"); "304.22" (ICD-9-CM: "Cocaine dependence, episodic"); "648.32" (ICD-9-CM: "Drug dependence of mother, delivered, with mention of postpartum complication"); "94.66" (ICD-9-CM: "Drug rehabilitation and detoxification"); "94.67" (ICD-9-CM: "Combined alcohol and drug rehabilitation"); "94.65" (ICD-9-CM: "Drug detoxification"); "94.69" (ICD-9-CM: "Combined alcohol and drug rehabilitation and detoxification"); "94.64" (ICD-9-CM: "Drug rehabilitation"); "94.68" (ICD-9-CM: "Combined alcohol and drug detoxification"); "HZ2ZZZZ" (ICD-10-PCS: "Detoxification Services for Substance Abuse Treatment"); "HZ33ZZZ" (ICD-10-PCS: "Individual Counseling for Substance Abuse Treatment, 12-Step"); "HZ36ZZZ" (ICD-10-PCS: "Individual Counseling for Substance Abuse Treatment, Psychoeducation"); "HZ32ZZZ" (ICD-10-PCS: "Individual Counseling for Substance Abuse Treatment, Cognitive-Behavioral"); |

**Supplementary Table 2: Patient substance abuse summary. The hypothesis test used for categorical variables is chi-square and for continuous variables is t-test. SUD = substance use disorder. N = number of patients. % = percentage of patients.**

|  | No SUD | SUD | p-value |
| --- | --- | --- | --- |
| Total Subjects (n%) | 4706 (86.57) | 730 (13.43) |  |
|  |  |  |  |
| Cannabis related disorders (n%) | 0 (0.0) | 376 (51.5) | <0.001 |
| Other psychoactive substance related disorders (n%) | 0 (0.0) | 247 (33.8) | <0.001 |
| Opioid related disorders (n%) | 0 (0.0) | 219 (30.0) | <0.001 |
| Alcohol usage (n%) | 0 (0.0) | 167 (22.9) | <0.001 |
| Poisoning by adverse effect of and underdosing of narcotics and psychodysleptics, hallucinogens (n%) | 0 (0.0) | 129 (17.7) | <0.001 |
| Other stimulant related disorders (n%) | 0 (0.0) | 99 (13.6) | <0.001 |
| Sedative hypnotic or anxiolytic related disorders (n%) | 0 (0.0) | 89 (12.2) | <0.001 |
| Cocaine related disorders (n%) | 0 (0.0) | 77 (10.5) | <0.001 |
| Inhalant related disorders (n%) | 0 (0.0) | 27 (3.7) | <0.001 |
| Hallucinogen related disorders (n%) | 0 (0.0) | 14 (1.9) | <0.001 |
| Nicotine dependence (n%) | 0 (0.0) | 5 (0.7) | <0.001 |
| Abuse of non-psychoactive substances (n%) | 0 (0.0) | 2 (0.3) | 0.011 |

**Supplementary Table 3: Univariable and multivariable analysis for organ dysfunction in patients with and without history of SUD. This table is similar to Table 2, except it does not group “Asian”, “American Indian or Alaska Native”, and “Native Hawaiian or Other Pacific Islander” races into “Other”. SUD = substance use disorder. OR = Odds ratio. CI = confidence interval. SDOH = social determinants of health.**

|  | Univariable Analysis | | Multivariable Analysis | |
| --- | --- | --- | --- | --- |
|  | **OR (95% CI)** | **P-value** | **OR (95% CI)** | **P-value** |
| Sex (Ref: male) |  |  |  |  |
| Female | 0.64 (0.58-0.72) | ***<0.001*** | 0.65 (0.58-0.72) | ***<0.001*** |
|  |  |  |  |  |
| Race (Ref: White) |  |  |  |  |
| Black or African American | 1.07 (0.94-1.21) | 0.30 | 1.12 (0.99-1.27) | 0.07 |
| Asian | 0.90 (0.66-1.22) | 0.49 | 0.96 (0.70-1.31) | 0.79 |
| American Indian or Alaska Native | 0.58 (0.29-1.16) | 0.12 | 0.57 (0.28-1.15) | 0.11 |
| Native Hawaiian or Other Pacific Islander | 1.13 (0.41-3.12) | 0.81 | 1.18 (0.42-3.32) | 0.75 |
|  |  |  |  |  |
| Age |  |  |  |  |
| 1-year increase | 1.01 (0.99-1.03) | 0.24 | 1.00 (0.98-1.02) | 0.95 |
|  |  |  |  |  |
| Any pediatric complex chronic condition (Ref: Absence) |  |  |  |  |
| Presence | 0.91 (0.82-1.01) | 0.08 | 0.94 (0.84-1.04) | 0.24 |
|  |  |  |  |  |
| SDOH (Ref: Absence) |  |  |  |  |
| Presence | 1.03 (0.87-1.22) | 0.71 | 1.01 (0.85-1.20) | 0.93 |
|  |  |  |  |  |
| SUD (Ref: Absence) |  |  |  |  |
| Presence | 1.87 (1.59-2.2) | ***<0.001*** | 1.83 (1.55-2.17) | ***<0.001*** |
